# Breast Cancer: Molecular Pathogenesis, Targeted Therapy, Screening, and Prevention

**DOI:** 10.1002/mco2.70560

**Published:** 2026-01-07

**Authors:** Huijun Lei, Jinzhen Fu, Wei Gu, Hongjin Qiao, Huixue Guo, Zijian Chen, San Ming Wang, Tianhui Chen

**Affiliations:** ^1^ Department of Cancer Prevention, Zhejiang Cancer Hospital, Hangzhou Institute of Medicine (HIM) Chinese Academy of Sciences Hangzhou Zhejiang China; ^2^ Faculty of Health Sciences University of Macau Taipa Macau China; ^3^ Postgraduate Training Base Alliance of Wenzhou Medical University (Zhejiang Cancer Hospital) Wenzhou Zhejiang China; ^4^ Hangzhou Normal University Hangzhou Zhejiang China

**Keywords:** breast cancer, pathogenesis, targeted therapy, screening, prevention, risk assessment

## Abstract

Breast cancer is the most common cancer and the leading cause of cancer‐related death among women worldwide. Advances in molecular biology, high‐throughput sequencing, and integrative‐omics have deepened the understanding of its heterogeneity by clarifying mechanisms linked to genetic susceptibility, epigenetic regulation, oncogenic signaling, and immune evasion. Although those developments have driven progress in targeted therapy and screening, concerns on drug resistance, toxicity, global inequities, and suboptimal risk stratification continue to limit outcomes. This review systematically summarizes current advances across four interconnected areas of breast cancer research and management, including molecular pathogenesis, targeted therapy, screening, and prevention. It describes key biological processes that shape tumor heterogeneity and examines targeted therapies, including endocrine agents, HER2‐directed drugs, CDK4/6 and PI3K/AKT/mTOR inhibitors, antibody–drug conjugates, and immunotherapies, together with mechanisms of resistance and emerging treatment targets. It also evaluates evolving approaches in risk stratification and screening, highlighting progress in digital breast tomosynthesis, magnetic resonance imaging, contrast‐enhanced mammography, and artificial intelligence‐assisted interpretation. By integrating cutting‐edge molecular insights with clinical advances, this review further highlights the expanding opportunities for personalized therapy and precision prevention. It outlines future directions linking multiomics and artificial intelligence to more equitable and effective breast cancer management.

## Graphical abstract

The review manuscript examines the molecular pathogenesis, screening, prevention advances, and evolving targeted therapies in breast cancer. It highlights the integration of AI and multiomics for precision prevention and personalized treatment, pointing toward future directions in equitable cancer care.

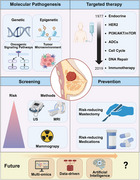

## 1 Introduction

Breast cancer is the most common cancer and the leading cause of cancer‐related mortality among women worldwide, imposing a significant economic and social burden on healthcare systems [1]. Recent data show a continuing increase in breast cancer incidence [2], driven in part by improved screening capabilities [3]. In 2022, approximately 2.3 million new cases and 670,000 deaths were reported globally, accounting for 25% of new cancer diagnoses and 15.5% of cancer deaths among women. Globally, breast cancer accounts for one in four female cancers and one in six female cancer‐related mortality [1]. By 2050, projected incidence shall increase by 38% while mortality shall increase by 68%, with the heaviest burden expected in countries with low scores of human development index scores [4].

Breast cancer is a heterogeneous disease encompassing multiple subtypes with distinct molecular and clinical features. Current clinical classification of breast cancer relies largely on the use of immunohistochemistry (IHC) markers of estrogen receptor (ER), progesterone receptor (PR), human epidermal growth factor receptor 2 (HER2), and Ki‐67 to classify breast cancer into four subtypes, that is, Luminal A (ER+ and/or PR+, HER2−, low Ki‐67), Luminal B (ER+, HER2±, high Ki‐67), HER2‐enriched (ER−, PR−, HER2+), and triple‐negative breast cancer (TNBC). These subgroups differ in prognosis, biology, and therapeutic response [5]. However, the field is moving rapidly beyond IHC‐based classification toward high‐resolution, omics‐driven system that captures tumor complexity at genomic, transcriptomic, and proteomic levels [6, 7]. Single‐cell RNA sequencing (scRNA‐seq) further delineates intratumoral heterogeneity, identifying rare cellular subpopulations implicated in resistance and metastasis [8]. Together, these advances underpin a more nuanced molecular taxonomy that informs therapeutic development.

Breast cancer has also been a typical example for precision medicine. Tailored treatment according to tumor biology has yielded major therapeutic advances and established new clinically meaningful subtypes. For example, trastuzumab deruxtecan (T‐DXd), an antibody–drug conjugate, has extended the HER2‐directed therapy into the HER2‐low setting, conferring significant survival benefit and reshaping the therapeutic paradigm [9, 10]. Similarly, the CAPItello‐291 trial demonstrated that the AKT inhibitor capivasertib combined with fulvestrant improves progression‐free survival (PFS) in patients with hormone receptor (+) HER2(−) tumors harboring PIK3CA, AKT1, or PTEN alterations [11, 12]. TNBC was considered uniformly aggressive, however, the KEYNOTE‐355 trial showed that pembrolizumab plus chemotherapy extended survival in patients with programmed death‐ligand 1 (PD‐L1)‐high tumors [13]. These successes highlight the causal link between molecular drivers and therapeutic benefit, affirming that precision medicine is now a clinical reality rather than a theoretical consideration. Comprehensive molecular profiling is likely to become routine in the near future as the current ER, PR, and HER2 testing.

A number of risk factors for breast cancer have been identified, for example, genetics, family history, personal history of breast disease, radiation, and dense breast [14, 15, 16, 17, 18]. As these risk factors are rarely amenable to modification through intervention, the primary objective of breast cancer prevention and control is the early detection and treatment of the disease. The use of screening mammograms prior to the onset of symptoms allows for the early detection of breast cancer [19, 20], thereby reducing mortality and the incidence of advanced cancer. Breast cancer screening serves as a fundamental component of preventive healthcare. In recent decades, numerous developed countries are practicing population‐based breast cancer screening programs [21, 22, 23], whereas in many low‐ and middle‐income countries, lack of resources hampers the implementation of mass screening programs. The mortality rate of breast cancer is higher and the disease is often diagnosed at later stage for women in those countries, compared with women in high‐income countries [24].

The principles of precision medicine are now extending into prevention and screening. Risk assessment models are being refined to support tailored interventions. While the traditional tools such as the Gail model demonstrate variable accuracy across populations, the approach of polygenic risk score (PRS) enable more granular lifetime risk estimation, guiding decisions on enhanced screening or preventive therapy [25]. Screening technologies are also evolving. While digital breast tomosynthesis (DBT) and magnetic resonance imaging (MRI) offer superior diagnostic accuracy [26], and artificial intelligence (AI)‐assisted image interpretation has shown to increase cancer detection rates by 13–18% [27, 28, 29]. Nonetheless, concerns on efficacy, risks, cost effectiveness and inconsistency among different guidelines (e.g., starting age at screening for women with average risk) may hinder widespread implementation.

This review critically appraises four domains central to contemporary breast cancer management: molecular pathogenesis, targeted therapy, screening technologies, and prevention strategies. Advances in these areas are promoting deeper understanding cancer heterogeneity, propelling integration of precision medicine into routine care, transforming therapeutic potentials, reshaping the risk assessment, screening, and prevention of breast cancer.

## 2 Molecular Pathogenesis of Breast Cancer

**FIGURE 1:**
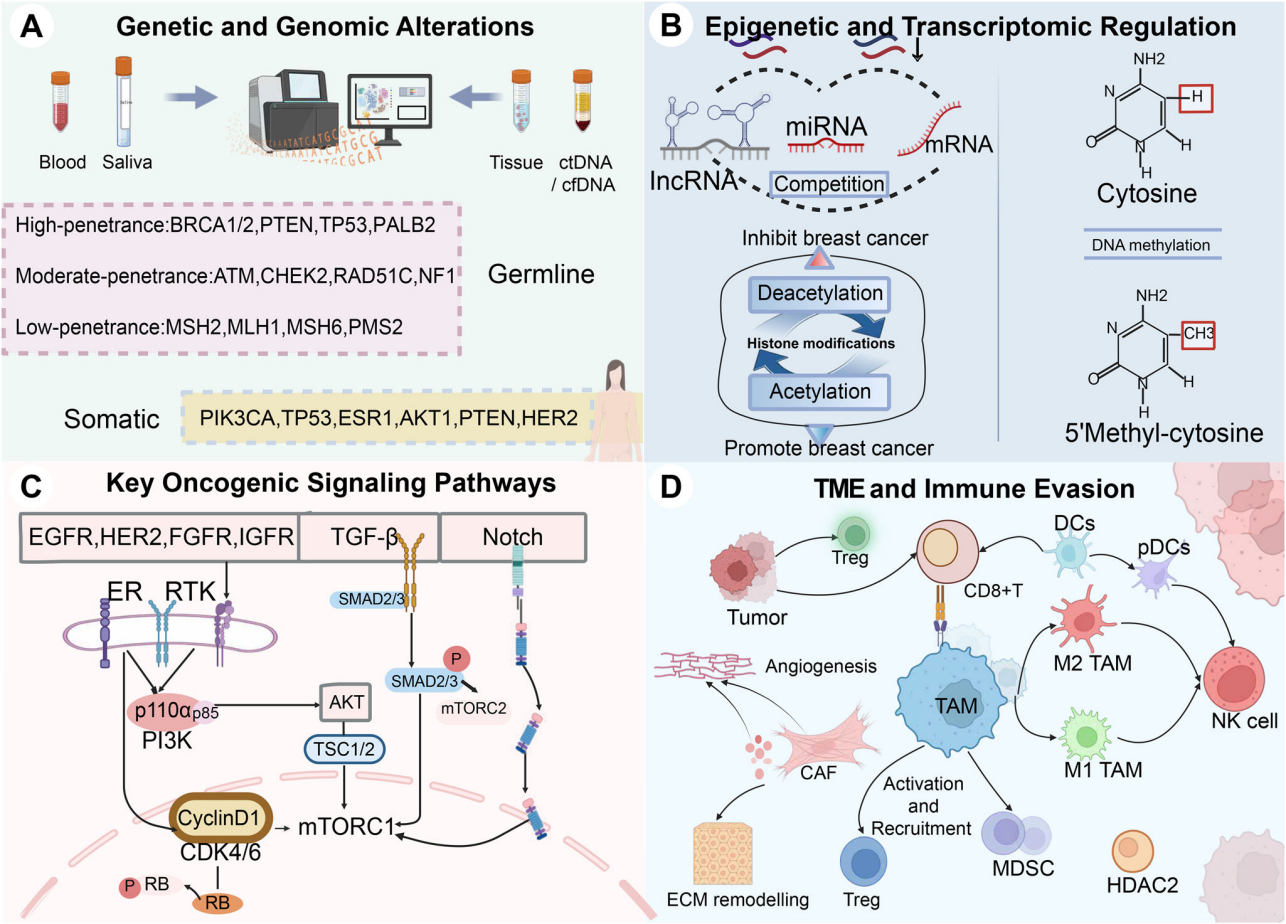
Key molecular mechanisms in the development and progression of breast cancer. The figure summarizes four major mechanisms that drive breast cancer initiation and progression: (A) genetic and genomic alterations; (B) epigenetic and transcriptomic regulation; (C) key oncogenic signaling pathways; (D) tumor microenvironment and immune evasion.

The initiation and progression of breast cancer are driven by a complex interplay of genetic susceptibility, hormonal dysregulation, epigenetic and transcriptomic alterations, immune dysregulation, and interactions with the tumor microenvironment (TME) (Figure 1). These processes drive genomic instability and clonal evolution, leading to marked cellular subtype differentiation and variability in therapeutic responses among breast cancers [30]. With the rapid advancement of multiomics technologies, including single‐cell transcriptomics, spatial transcriptomics, and circulating cell‐free DNA sequencing, our understanding of breast cancer pathogenesis is shifting from static, single‐factor models to dynamic, systems‐level network frameworks [7, 31]. This paradigm provides a theoretical foundation to refine molecular classification and develop individualized treatment and early intervention strategies.

### 2.1 Genetic Mechanism

Germline mutation constitutes a significant genetic basis of breast cancer susceptibility, particularly in patients with early‐onset, family history, or bilateral breast cancer. Among them, pathogenic variants in the *BRCA1* and *BRCA2* genes are the most prevalent and the major risk factor for hereditary breast and ovarian cancer syndrome (HBOC) [32]. These two genes play critical roles in repairing double‐strand DNA breaks through the DNA homologous recombination repair pathway, and their functional defects lead to increased genomic instability and elevated risk of breast and ovarian cancers by approximately 60% [33]. In addition to *BRCA1/2*, other genes such as *PALB2*, *BARD1*, *PTEN*, *TP53*, *ATM*, and *CHEK2* are also associated with elevated breast cancer risk [34]. Recent studies have shown that germline mutations in these genes are not only associated with breast cancer risk but also implicated in distant metastasis, as exemplified by *PCSK9* [35].

Somatic mutation is a direct driver of malignant transformation and progression of breast cancer cells. Somatic mutation in *TP53* is the most common genetic alterations in tumors, occurring in over 30% of breast cancers, typically arising in the early stages of tumorigenesis. These mutations are linked to a more aggressive disease course and poorer overall survival (OS), particularly prevalent in TNBC [36]. Somatic mutation in *PIK3CA* is another frequent somatic alteration in breast cancer, with a prevalence of up to 40% in ER(+) disease [37]. *PIK3CA* mutation leads to the abnormal activation of the PI3K/AKT/mTOR signaling pathway, which plays a central role in cell proliferation, survival, invasion, migration, apoptosis, glucose metabolism, and DNA repair [37]. The *ESR1* gene encodes ER‐α, which is a key driver in ER(+) breast cancer. Somatic mutation in *ESR1* mainly occurs in patients who relapse or develop metastases after endocrine therapy. The mutation enables ligand‐independent activation of the ER signaling pathway, thereby resulting in endocrine resistance [38].

Certain gene mutations not only serve as molecular hallmarks of tumorigenesis but also are actionable therapeutic targets. For instance, defined targeted treatment strategies have been developed for the patients with mutations in *PIK3CA*, *BRCA1/2*, and *ESR1*. *PIK3CA*‐mutated breast cancer can be treated with the PI3Kα‐specific inhibitor alpelisib, which has been approved by the United States Food and Drug Administration (US FDA) for ER(+)/HER2(−) advanced disease [39]; patients with germline *BRCA1/2* mutations benefit from poly(ADP‐ribose) polymerase (PARP) inhibitors such as olaparib or talazoparib [40]. Moreover, *ESR1* mutation suggests endocrine therapy failure, thus necessitating the use of selective ER degraders, such as fulvestrant or the combination of CDK4/6 inhibitors (CDK4/6i) to overcome drug resistance [41, 42].

Nevertheless, the pronounced molecular heterogeneity of breast cancer remains a major obstacle in elucidating its pathogenesis and guiding treatment strategies, functional annotation of mutations in noncoding regions remains insufficient, and integrative analyses of multiomics data face challenges of standardization, reproducibility, and interpretation. Therefore, future research will require the combination between the use of systems biology approaches and functional validation to identify truly pathogenic and druggable variants, thereby providing a more precise molecular foundation for dynamic monitoring of breast cancer development and individualized treatment.

### 2.2 Epigenetic Regulatory Mechanisms

Unlike genetic mutations, epigenetic regulation is reversible [43]. Epigenetic regulation modifies cellular functions by influencing gene expression without altering the DNA sequence, and it plays a pivotal role in the initiation and progression of breast cancer [44]. DNA methylation (DNAme), histone modifications, and chromatin remodeling can reprogram gene expression, while noncoding RNAs (ncRNAs) regulate tumor cell proliferation, metastasis, and drug resistance through complex networks [45].

DNAme refers to the covalent methylation of cytosine residues in DNA molecules, occurring mainly at CpG dinucleotide sites [46]. In breast cancer, abnormal DNAme manifests as promoter hypermethylation of tumor suppressor genes and hypomethylation of oncogenes or repetitive sequences, where hypermethylation leads to gene silencing and hypomethylation results in gene activation or genomic instability [46, 47]. The unbalanced methylation is a common alteration across multiple molecular subtypes of breast cancer and is particularly pronounced in TNBC [48]. The WID‐BC index, developed based on DNAme patterns in cervical samples, has been shown to effectively identify women at high risk of breast cancer, highlighting the potential of DNAme as a key molecular biomarker for early diagnosis and prognosis [49]. Moreover, analysis of DNAme profiles has enabled the classification of TNBC into two distinct epigenetic subtypes, the basal‐like and nonbasal‐like, providing novel insights into the epigenetic underpinnings of TNBC and potential targets for developing novel therapies [50].

Histone modification includes acetylation and methylation, which influence transcriptional activity through regulating chromatin structure [51]. Histone acetylation is dynamically regulated by the balanced histone acetyltransferases and histone deacetylases (HDACs). In breast cancer, aberrant overexpression of HDACs leads to histone deacetylation, resulting in condensed chromatin and gene silencing, thereby suppressing tumor suppressor gene expression [52]. HDAC inhibitors have shown therapeutic potential in preclinical and clinical trials, especially when combined with immunotherapy, as they can remodel the TME, overcome immune evasion, and have emerged as potential targets for breast cancer therapy [53]. Dysregulation of histone methyltransferases (HMTs) and demethylases is widespread in breast cancer. EZH2, a histone H3K27 methyltransferase, promotes the initiation and progression of breast tumors by epigenetically regulating the Wnt and mTORC1 signaling pathways [54]. NSD2/WHSC1, an H3K36me2 methyltransferase, has been shown to promote TNBC metastasis by activating ULK1‐dependent autophagy. This finding revealed a novel mechanism of NSD2 in TNBC metastasis and provided NSD2 as a potential therapeutic target [55].

ncRNAs are a class of RNA molecules that do not encode proteins but play important roles in the regulation of gene expression. Among them, microRNAs (miRNAs) and long ncRNAs (lncRNAs) are the two most widely studied types [56]. miRNAs are short RNA molecules of approximately 20–25 nucleotides in length, which bind to the 3′ untranslated regions of target mRNAs to inhibit their translation or promote their degradation, thereby negatively regulating gene expression [57]. In breast cancer, many miRNAs are aberrantly expressed, including both oncogenic miRNAs and tumor‐suppressive miRNAs [57,^58^]. lncRNAs are ncRNAs longer than 200 nucleotides, which regulate gene expression through multiple mechanisms, including chromatin remodeling, transcriptional regulation, posttranscriptional regulation, and acting as miRNA sponges [59]. In breast cancer, numerous lncRNAs have been found to be aberrantly expressed and involved in tumor initiation and progression. For example, lncRNA NAMPT–AS is upregulated in TNBC and promotes tumor progression and metastasis by epigenetically activating NAMPT expression [60]. Complex interaction networks exist between miRNAs and lncRNAs. Many lncRNAs can act as competing endogenous RNAs, competitively binding to miRNAs and thereby relieving their suppression of target mRNAs, indirectly upregulating the expression of target genes [61].

Traditional transcriptomic studies are usually based on the average expression profiles of large numbers of cells, which obscure intra‐tumoral cellular heterogeneity. Single‐cell and spatial transcriptomics technologies provide new opportunities to uncover the epigenetic and transcriptomic heterogeneity of breast cancer. Recent studies integrating DNAme data with spatial transcriptomics have demonstrated that epigenetic regulation underlies cellular state transitions in highly heterogeneous subtypes such as metaplastic breast carcinoma [62]. In addition, multiomics investigations combined with functional analyses have identified a novel chromatin remodeling complex, ARID1B–SMARCC2/SMARCB1, which suppresses epigenetic modification and drives malignant progression of TNBC [63]. These findings emphasize that multiomics approaches can uncover convergent epigenetic mechanisms governing tumor plasticity and progression, therefore, providing potential entry points for the development of epigenetic‐targeted therapies.

However, the diversity nature of epigenetic regulation makes it difficult to identify universal therapeutic targets. Because epigenetic modifications are highly dynamic, plastic, and variable among different breast cancer subtypes and even within an individual tumor, it remains a challenge to develop epigenetic‐targeted therapies that are universally effective for all patients. Although single‐omics technologies have achieved significant progress, it remains a formidable task to integrate epigenetic information of DNAme, histone modifications, and ncRNAs together with gene expression data, to construct comprehensive regulatory networks and uncover their synergistic roles in breast cancer pathogenesis. This task requires more advanced bioinformatics tools and computational modeling.

### 2.3 Key Oncogenic Signaling Pathways

Development and progression of breast cancer involve multiple signaling pathways. Aberrant activation or inactivation of these pathways can lead to uncontrolled cell proliferation, impaired apoptosis, and enhanced invasion and metastasis. The PI3K/AKT/mTOR signaling pathway is one of the most critical intracellular pathways. It plays key roles in cell proliferation, survival, metabolism, migration, and angiogenesis [37]. In breast cancer, this pathway is frequently hyperactivated due to PIK3CA mutations, loss or inactivation of the tumor suppressor PTEN, and hyperactivation of upstream receptor tyrosine kinases (RTKs) such as HER2 and epidermal growth factor receptor (EGFR) [37, 64]. Aberrant activation of the PI3K/AKT/mTOR pathway promotes tumor cell growth and survival, and is also strongly associated with the resistance to chemotherapy and endocrine therapy [38]. Combining with endocrine therapy, PI3K inhibitors alpelisib, AKT inhibitors capivasertib, and mTOR inhibitors everolimus have been applied clinically in breast cancer treatment, especially in hormone receptor (+)/HER2(−) subtypes, to overcome resistance [38, 64].

The ER signaling pathway is the central driver of hormone receptor (+) breast cancer, accounting for approximately 65–70% of all cases [65]. Estrogen binds to ER, induces ER dimerization and nuclear translocation, where it binds to estrogen response elements on DNA to regulate the transcription of its target genes, promoting proliferation and survival of breast cancer cells [38]. Endocrine therapy, including selective ER modulators (SERMs), aromatase inhibitors (AIs), and selective ER degraders (SERDs), suppress ER signaling by blocking estrogen‐ER binding or inhibiting estrogen synthesis. Endocrine therapy is the standard treatment for hormone receptor (+) breast cancer [38, 65].

The MAPK pathway, also known as the RAS–RAF–MEK–ERK pathway, plays essential roles in cell proliferation, differentiation, migration, and apoptosis. In breast cancer, aberrant MAPK pathway activation is often caused by overexpression of upstream RTKs (e.g., EGFR, HER2) or mutations in oncogenes such as RAS and BRAF [66]. Sustained activation of the MAPK pathway promotes malignant proliferation and invasion of tumor cells and is associated with resistance to endocrine therapy and chemotherapy. lncRNAs have been identified as critical regulators of the PI3K/AKT, MAPK, and TGF‐β pathways, with many lncRNAs exerting oncogenic functions by enhancing activation of these signaling cascades [59].

The CDK4/6–Rb axis is a central regulator of the G1 to S phase cell cycle transition [67]. In breast cancer, ERɑ overactivation is a common oncogenic feature, leading to phosphorylation and inactivation of RB protein, thereby releasing E2F transcription factors to promote cell cycle progression and proliferation [68]. Combination of CDK4/6i with endocrine therapy has significantly improved PFS in patients with hormone receptor (+)/HER2(−) advanced breast cancer by effectively targeting dysregulated cell cycle progression [69, 70, 71, 72]. Evidence indicates that the CDK4/6i abemaciclib promotes lysosomal degradation of B7‐H4, which in turn mitigates B7‐H4‐mediated immunosuppression, suggesting that CDK4/6i may exert immunomodulatory effects and open perspectives for combination with immunotherapy [73].

In addition, several other signaling pathways have recently attracted attention. For example, RANK signaling can upregulate stemness‐associated genes through the NF‐κB pathway and drive the expansion of breast cancer stem cell populations. RANK plays a dual role in the breast cancer signaling network, acting as a suppressor of early tumor initiation while accelerating malignant progression after tumor formation [74]. In solid tumors, cancer stem cell was first identified and isolated in breast cancer, and it plays critical roles in tumor initiation and progression. Kita‐Kyushu lung cancer antigen‐1 (KK‐LC‐1) has recently been identified as a novel marker of TNBC stem cells, and the “KK‐LC‐1–FAT1–Hippo–YAP–ALDH1A1” axis plays a central role in regulating TNBC stem cells. These offer breast cancer stem cells as potential new targets [75].

Intracellular signaling pathways in breast cancer do not function in isolation but constitute a highly complex network with extensive crosstalk and feedback regulation. When targeting a single signaling pathway, tumor cells may activate alternative bypass or downstream pathways to evade therapy, leading to drug resistance or substantially reducing treatment responses. Although many critical oncogenic pathways have been identified, predicting individual patient responses to specific pathway inhibitors remains challenging. Future research will require the development of more sensitive and specific biomarkers, combined with noninvasive approaches such as liquid biopsy, to enable dynamic monitoring of pathway activity and parallel evaluation of multiple signaling cascades. The development of tools capable of simultaneously assessing multiple pathway activities and guiding treatment decisions remains a critical challenge.

### 2.4 TME and Immune Evasion

TME is a complex ecological system on which tumor cells depend for survival, consisting of tumor cells, immune cells, stromal cells, blood vessels, lymphatic vessels, and extracellular matrix, among others [76]. The composition and function of the TME have a decisive impact on the progression, metastasis, and therapeutic response of breast cancer. Tumor immune evasion is a key mechanism by which tumor cells escape immune surveillance and represents a major obstacle to immunotherapy.

The TME contains diverse immune cell populations, including tumor‐associated macrophages (TAMs), T cells, B cells, natural killer (NK) cells, myeloid‐derived suppressor cells (MDSCs), and dendritic cells, among others [77]. These immune cells play dual roles in tumor progression, exerting both suppressive and promotive effects, as described by the concept of “cancer immunoediting” [78]. TAMs are among the most abundant immune cells in the TME, and their infiltration levels are closely correlated with breast cancer prognosis. Increased M2‐type TAMs often indicate higher grade and poorer survival outcomes [79]. In addition, tumor‐infiltrating lymphocytes, particularly CD8+ T cells, are generally associated with a favorable prognosis. However, TME in breast cancer are often functionally impaired due to suppression by immune checkpoint pathways such as programmed cell death protein 1 (PD‐1)/PD‐L1 [80]. At the same time, immunosuppressive cytokines play crucial roles in breast cancer. For example, IL‐10, TGF‐β, and IL‐35 are widely present in the TME, where they inhibit antitumor immune responses and recruit additional immunosuppressive cells into the TME [81]. Notably, IL‐35 produced by regulatory T cells (Tregs) and breast cancer cells can convert conventional T cells into immunosuppressive Tregs and suppress the secretion of proinflammatory cytokines, thereby facilitating immune evasion [82]. Immune cells and cytokines in breast cancer act synergistically within the TME. Cancer‐associated fibroblasts (CAFs) are the most abundant stromal cell type in the TME and constitute a key component promoting tumor growth, invasion, metastasis, and angiogenesis. On the one hand, CAFs remodel the extracellular matrix, secreting collagen, fibronectin, and other matrix proteins to construct a scaffold that facilitates tumor cell adhesion and migration, thereby establishing a “tumor‐friendly” stromal environment [83]. On the other hand, CAFs secrete cytokines, growth factors, and various proangiogenic mediators, inducing proliferation of neighboring endothelial cells and promoting neovascularization, which provides a continuous supply of oxygen and nutrients to breast cancer [84]. Vascular endothelial growth factor (VEGF) is the major angiogenic factor and is frequently overexpressed in breast cancer. Under hypoxic conditions, CAFs upregulate VEGF expression through HIF‐1α signaling, further promoting angiogenesis [85, 86]. Aberrant tumor vasculature supplies oxygen and nutrients for metastatic dissemination. CXCL12 secreted by breast cancer CAFs markedly enhances vascular permeability and neovascular formation, facilitating cancer cell intravasation and thus promoting metastasis [87]. Based on these mechanisms, the proangiogenic function of CAFs not only supports the acquisition of sufficient oxygen and nutrients by tumor cells but also provides both structural and signaling support for vascular infiltration and metastatic spread.

Immune evasion in breast cancer results from the combined effects of cellular components, molecular signaling, and the metabolic milieu through diverse mechanisms. First, tumor cells and immunosuppressive cells in the TME (such as Tregs and MDSCs) can highly express immune checkpoint molecules such as PD‐L1 and CTLA‐4. These molecules bind to corresponding receptors on T cells, suppressing their activation and function, thereby leading to T‐cell exhaustion and immune suppression [88, 89]. For example, HDAC2 can upregulate PD‐L1 expression and enhance immune evasion by suppressing T‐cell activity [90]. Breast cancer cells also secrete various chemokines to recruit immunosuppressive cells such as Tregs, MDSCs, and M2‐type TAMs into the TME, thereby establishing an immunosuppressive microenvironment [81]. Moreover, breast cancer stem cells mediate tumor metabolic reprogramming by secreting macrophage migration inhibitory factor, thereby driving immune evasion [91]. Migration inhibitory factor activates the WNT/β‐catenin signaling pathway and upregulates c‐MYC‐mediated transcription of the glycolytic enzyme aldolase C, thereby promoting glycolysis, enhancing tumor growth and metastasis, and driving immune evasion [91].

Single‐cell and spatial omics technologies have made remarkable progress in revealing TME heterogeneity. These approaches can characterize the molecular features, spatial distribution, and interactions of different cell types within the TME, providing a more comprehensive understanding of how the TME promotes tumor progression and immune evasion [92, 93]. Using single‐cell and spatial transcriptomics, recent work has shown that breast cancer cells secrete arginine to drive polyamine biosynthesis and TDG‐mediated epigenetic reprogramming in TAMs, which polarizes them toward a protumor immunosuppressive phenotype and suppresses CD8+ T‐cell activity, thereby playing a critical role in immune evasion [94].

Despite growing insights into the TME and immune evasion, significant challenges remain. The microenvironment of metastatic lesions differs markedly from that of primary tumors, which explains why immunotherapies effective against primary sites often show limited efficacy in metastases. Understanding site‐specific characteristics of the TME and developing tailored immunotherapeutic strategies will be a key to overcome the clinical barriers in treating metastatic breast cancer.

## 3 Targeted Therapies for Breast Cancer

**FIGURE 2:**
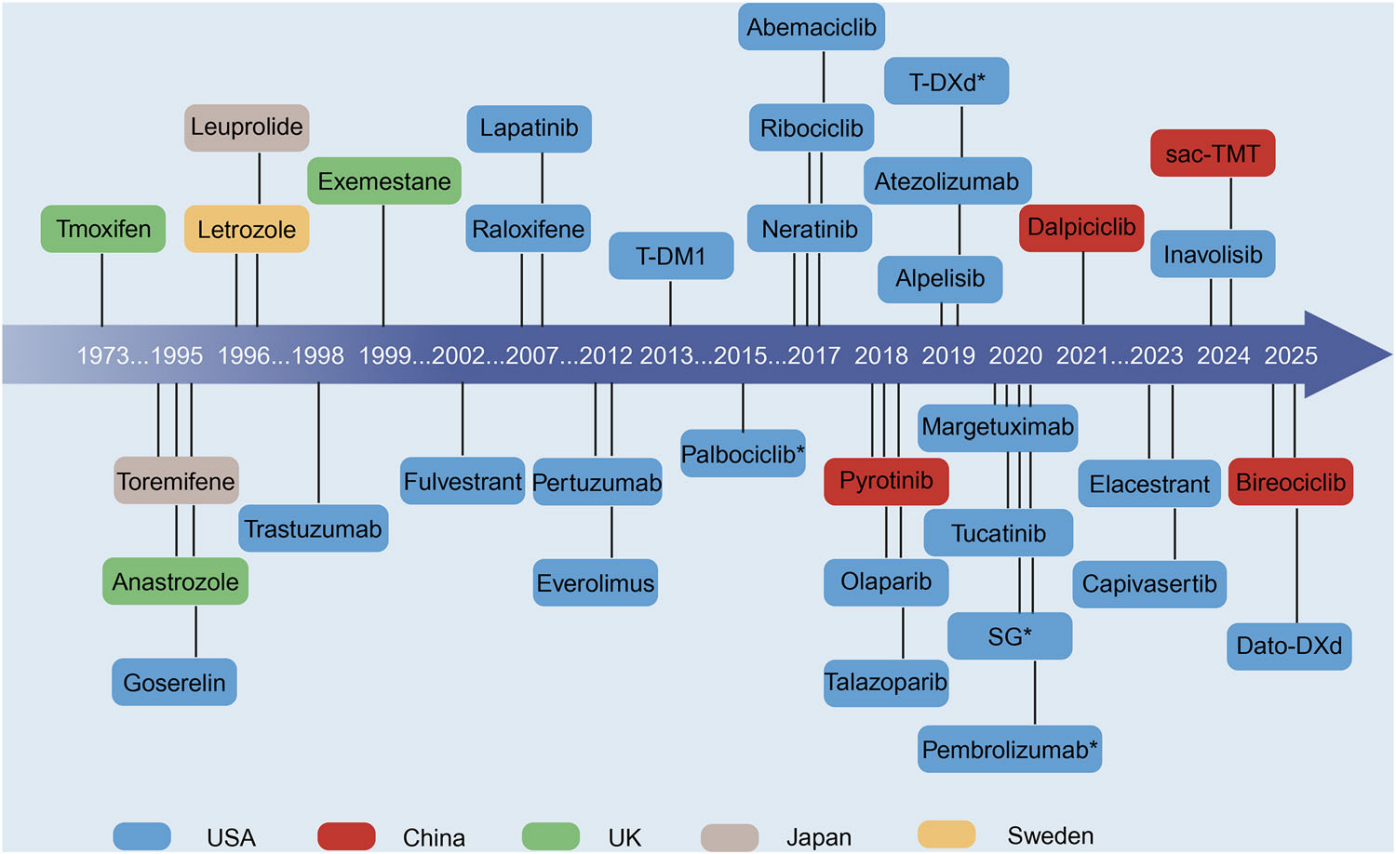
Timeline of approval for targeted therapeutic agents in breast cancer. Approved agents are positioned along a horizontal timeline according to their initial regulatory approval year(s). (*Drugs received US FDA's “accelerated approval”; T‐DM1, trastuzumab emtansine; T‐DXd, trastuzumab deruxtecan; SG, sacituzumab govitecan; Dato‐DXd, datopotamab deruxtecan; sac‐TMT, sacituzumab tirumotecan).

Since the approval of tamoxifen in the 1970s, targeted therapies for breast cancer have advanced over five decades (Figure 2). Currently, at least 35 reagents have been approved worldwide, involving endocrine therapies, anti‐HER2 drugs, CDK4/6i, PI3K/AKT/mTOR inhibitors, antibody–drug conjugates (ADCs), and immunotherapies. By interfering with specific molecular drivers or the TME, these therapies suppress tumor growth and improve survival. In this section, we summarize and discuss the major classes of targeted drugs, outlining their development, clinical impact, and future directions.

### 3.1 Endocrine Therapy

Endocrine therapy for breast cancer inhibits tumor growth by blocking the stimulating effect of estrogen on cancer cells. It is the cornerstone of the treatment of hormone receptor‐positive breast cancer, which accounts for the majority of cases. Agents are broadly classified as SERMs, SERDs, and AIs, each with distinct mechanisms and clinical implications.

Tamoxifen, the prototypical SERM, has long been established as effective in both treatment and prevention of ER‐positive disease, including in pre‐ and postmenopausal women [95, 96]. Long‐term follow‐up has consistently demonstrated reduced recurrence, yet its use is offset by increased risks of endometrial cancer and venous thromboembolism [97, 98]. Recent evidence from the TAM‐01 trial suggests that low‐dose tamoxifen (5 mg daily for 3 years) significantly reduces recurrence of noninvasive disease with a more favorable safety profile, highlighting the need to balance efficacy against toxicity [99]. Two other SERMs, toremifene and raloxifene, also approved for the treatment of breast cancer have shown acceptable efficacy and tolerability in subsequent clinical trials [100, 101].

Fulvestrant is a SERD for endocrine therapy of advanced hormone receptor‐positive breast cancer that demonstrated superior PFS over anastrozole in the Phase III FALCON trial (median PFS 16.6 vs. 13.8 months) [102]. The final OS [median, 44.8 vs. 42.7 months; hazard ratio (HR) = 0.97; 95% confidence interval (CI) 0.77–1.21; *p* = 0.7579] analysis of the FALCON trial showed no significant difference between fulvestrant and anastrozole [103]. However, it is often accompanied by adverse reactions such as hot flushes and arthralgia [102]. These limitations have driven the development of next‐generation oral SERDs with improved bioavailability and convenience.

AIs act by inhibiting androgen to estrogen conversion. The third‐generation AIs commonly used in breast cancer treatment include anastrozole, exemestane, and letrozole. Due to the role of compensatory physiological regulation, these drugs are contra‐indicated for premenopausal women [104]. The ATAC trial compared the efficacy of anastrozole and tamoxifen in the adjuvant treatment of early breast cancer. The 10‐year follow‐up results showed that anastrozole was more effective and safer than tamoxifen in the long‐term adjuvant treatment of postmenopausal women with ER(+) and/or PR(+) early breast cancer [105]. Exemestane is often used as an alternative treatment for postmenopausal patients with advanced breast cancer after tamoxifen treatment [106]. In premenopausal women, AIs require concomitant ovarian function suppression (OFS) to be effective. Long‐term follow‐up of the SOFT and the TEXT trials demonstrated that OFS combined with either tamoxifen or exemestane significantly improved disease‐free survival (DFS) compared with tamoxifen alone, underscoring the clinical value of ovarian suppression in younger patients at higher risk [107]. Letrozole also has similar biological effects to anastrozole and exemestane. OFS can be achieved by gonadotropin‐releasing hormone (GnRH) agonists, ovariectomy, or ovarian radiotherapy. Common GnRH agonists include goserelin and leuprolide, which are commonly used in ER(+) and/or PR(+) premenopausal women with breast cancer. In the SOFT trial, premenopausal women were randomly assigned to receive 5 years of tamoxifen, tamoxifen combined with OFS, or exemestane combined with OFS [108]. After 12 years of long‐term follow‐up, the results showed that compared with tamoxifen monotherapy, OFS combined with tamoxifen had significant improvements in DFS (76.1 vs. 71.9%, HR = 0.82; 95% CI 0.69–0.98) and OS (89.0 vs. 86.8%, HR = 0.78; 95% CI 0.60–1.01) [108]. The result suggests the long‐term effectiveness of OFS in adjuvant endocrine therapy for premenopausal breast cancer.

The introduction of oral SERDs represents a major therapeutic advance. Elacestrant is currently the only oral SERD approved for use in ER(+) metastatic breast cancer combined with *ESR1* mutated patients [109]. Improved PFS was observed with Elacestrant compared with standard endocrine monotherapy in patients who progressed after CDK4/6i in the Phase III EMERALD trial, particularly those with *ESR1* mutations [110]. Camizestrant is a next‐generation oral SERD. The latest SERENA‐6 trial data showed that first‐line camizestrant plus a CDK4/6i significantly prolongs median PFS (16.0 vs. 9.2 months, HR = 0.44; 95% CI 0.31–0.60; *p* < 0.0001) compared with continued AIs‐based combination therapy in patients with newly detected *ESR1*‐mutant, ER(+)/HER2(−) advanced breast cancer [42]. Other agents, such as giredestrant and imlunestrant, are under active Phase III investigation (Table 1). Giredestrant and imlunestrant are two additional oral SERDs currently under investigation. They both act by binding to the ER and triggering its degradation. Although the Phase II acelERA BC trial of giredestrant missed statistical significance for its primary endpoint of investigator‐assessed PFS, a favorable benefit trend was observed in patients with *ESR1*‐mutant tumors, the finding that provides a strong rationale for the ongoing Phase III programme [111]. The Phase III EMBER‐3 trial demonstrated that imlunestrant monotherapy significantly prolonged PFS versus standard‐of‐care in patients harboring *ESR1* mutations, and that the imlunestrant–abemaciclib combination improved PFS irrespective of *ESR1* mutation status [112]. Novel oral SERDs, which are currently a major focus of breast‐cancer drug development, illustrate a paradigm shift. But their ultimate place in therapy will depend on whether PFS gains translate into durable survival improvements and whether tolerability profiles support long‐term use.

**TABLE 1:** Emerging target drugs for breast cancer.

Target molecule	Agents	Mechanism	Current clinical trial stage	
			Ongoing trials, Phase	Ongoing/all
ER	Lasofoxifene	In bone tissue: activating ER, suppressing osteoclasts, and stimulating osteoblasts In breast tissue: binding to the ER and blocks the estrogen signaling pathway (especially in ESR1 mutant ER+ breast cancer)	ELAINE I (NCT03781063), II ELAINE III (NCT05696626), III	SERMs: 2/3
	Camizestrant (AZD9833)	Binding to the ER and induces a conformational change that results in the degradation of the receptor	CAMBRIA‐2 (NCT05952557), III CAMBRIA‐1 (NCT05774951), III EvoPAR‐BR01 (NCT06380751), III SERENA‐1 (NCT03616587), I SERENA‐2 (NCT04214288), II SERENA‐4 (NCT04711252), III SERENA‐6 (NCT04964934), III	Oral SERDs: 21/27
	Giredestrant (GDC‐9545)	Binding to the ER, induces a conformational change that facilitates ubiquitin–ligase recognition and tagging of the receptor, leading to ER protein degradation via the proteasomal pathway	NCT03332797, Ia/Ib NCT07100106, Ib/II Neo‐AGILE (NCT06259929), II PREcoopERA (NCT05896566), II NCT04576455, II NCT04961996, III NCT04546009, III NCT05296798, III NCT06065748, III NCT05306340, III	
	Imlunestrant (LY3484356)	Competitively binding the ER, thereby inducing conformational changes and subsequent degradation of ER.	NCT05509790, I EMBER (NCT04188548), I EMBER‐3 (NCT04975308), III EMBER‐4 (NCT05514054), III	
	Vepdegestrant (ARV‐471)	Harnessing the ubiquitin–proteasome system to induce direct ubiquitination and subsequent degradation of ER	TACTIVE‐E (NCT05501769), I NCT04072952, I/II NCT06206837, Ib/II TACTIVE‐U:Sub‐Study A (NCT05548127), Ib/II TACTIVE‐U:Sub‐Study B (NCT05573555), Ib/II TACTIVE‐U:Sub‐Study C (NCT06125522), Ib/II NCT05549505, II VERITAC‐2 (NCT05654623), III VERITAC‐3 (NCT05909397), III	PROTAC: 9/11
	Palazestrant (OP‐1250)	Blocking both transcriptional activation function domains, AF1 and AF2, required for the estradiol‐generated transcriptional activity of ER	NCT05266105, I NCT05508906, Ib OPERA‐01 (NCT06016738), III OPERA‐02 (NCT07085767), III	CERAN: 4/5
	HRS8807	Binding to the ER, inhibiting both ligand‐dependent and potentially ligand‐independent signaling pathways		SERCA: 2/4
	H3B‐6545	Inactivating both wild‐type and mutant ERα by targeting C530 and enforcing antagonist conformation	NCT04568902, I NCT04288089, I	
GnRH receptor	Triptorelin	First stimulating and then suppressing the pituitary–gonadal axis, achieving pharmacological castration	SOLTI‐2104 (NCT05982093), II ESCALATE (NCT06225284), II OFSET (NCT05879926), III ProFertil (NCT05328258), III	OFS: 4/24
HER‐2	BNT‐323	Involving an antibody that binds to HER2 receptors on cancer cells, delivering a topoisomerase‐1 inhibitor payload into the cell to destroy it	NCT06827236, I/II	HER2 mAb: 1/1
	BNT‐323/DB‐1303	An ADC that targets HER2‐expressing cancer cells by using a humanized monoclonal antibody to deliver a topoisomerase I inhibitor drug directly into the cancer cells, leading to their destruction	NCT05150691, I/IIa Dynasty‐Breast01 (NCT06265428), III Dynasty‐Breast02 (NCT06018337), III	ADCs(HER2‐targeted): 50/60
	FS‐1502	A HER2‐targeting ADC comprising a cancer‐selective cleavable β‐glucuronide linker, an anti‐HER2 antibody derived from trastuzumab, and an antimitotic agent, monomethyl auristatin F (MMAF), which inhibits tubulin polymerization	NCT05755048, III	
	ARX‐788	An ADC consisting of a HER2‐targeting antibody site‐specifically conjugated with a potent antitubulin cytotoxic drug‐linker, AS269; The site‐specific conjugation is achieved by first incorporating the nonnatural amino acid, para‐acetyl phenylalanine (pAF), into the antibody, followed by covalent conjugation of AS269 to the pAF to form a highly stable oxime bond resulting in a DAR 2 ADC.	NCT06578286, II ACE‐Breast‐03 (NCT04829604), II NCT04983121, II NCT06224673, II NCT06663748, II NCT05426486, II/III	
	Zanidatamab (ZW25)	Binding to HER2 across a range of expression levels, formation of receptor clusters, and receptor internalization resulting in HER2 downregulation, inhibits growth factor‐dependent and growth factor‐independent tumour‐cell proliferation and activates immune‐mediated responses.	EmpowHER 208 (NCT07102381), II DiscovHER PAN‐206 (NCT06695845), II NCT05035836, II NCT06435429, III	
	SHR‐A1811	Comprising a humanized HER2‐directed mAb (trastuzumab) conjugated to DNA topoisomerase I inhibitors (SHR169265) via cleavable tetrapeptide‐based linkers	NCT07177950, I/II NCT05911958, II NCT07129187, II NCT06788197, Ib/II NCT05353361, Ib/II NCT05845138, Ib/II NCT07111832, III NCT06126640, III … A total of 36 trials	
	ZN‐A‐1041	Binding to and inhibit the HER2 protein. By blocking HER2's activity, it inhibits tumor cell growth.	NCT05593094, I	TKIs: 1/2
CDK2/4/6	RGT‐419B	Targeting and inhibiting CDK2, CDK4, and CDK6, which inhibits the phosphorylation of retinoblastoma protein (Rb) early in the G1 phase and prevents CDK‐mediated G1‐S‐phase transition.	NCT06299124, I NCT05304962, I NCT07100106, Ib/II	CDKI: 15/19
CDK4/6	Trilaciclib	A short acting inhibitor of CDK4/6; trilaciclib causes transient G1 cell cycle arrest in hematopoietic cells protecting them against the myelosuppression caused by cytotoxic antineoplastic medications.	NCT05862610, II NCT06955156, II NCT06297811, II SMA‐BC‐002 (NCT05978648), II ToPCourT (NCT06027268), II	
CDK4	Atirmociclib (PF‐07220060)	Targeting the CDK4 kinase, rendering the retinoblastoma (Rb)/E2F transcription system inactive, which ultimately leads to cell cycle arrest in the G1 phase	NCT04557449, I NCT05262400, Ib/II NCT06206837, Ib/II NCT06465368, II FOURLIGHT‐1 (NCT06105632), II FourLight‐3 (NCT06760637), III	
CDK2	Tegtociclib (PF‐07104091)	Selectively binding to, and inhibiting the activity of CDK2, which may lead to cell cycle arrest through reduced phosphorylation of Rb, and other phosphotargets	NCT04553133, I/IIa NCT05262400, Ib/II	
PARP1/2/3	Rucaparib (AG014699)	Inhibiting PARP1, PARP2, and PARP3; inhibiting PARP traps the enzyme on damaged DNA, halting the repair process and forming toxic PARP–DNA complexes		PARPi: 14/58
PARP1/2	Veliparib (ABT‐888)	Working by inhibiting PARP enzymes, particularly PARP1 and PARP2; this inhibition causes an accumulation of DNA damage in cancer cells, leading to cell cycle arrest and apoptosis (cell death).	NCT01351909, I NCT01251874, I	
PARP1	Saruparib (AZD5305)	Selectively targeting and binding to PARP and prevents PARP‐mediated DNA repair of single‐strand DNA breaks via the base–excision repair pathway; this enhances the accumulation of DNA strand breaks and promotes genomic instability and eventually leads to apoptosis.	PETRA (NCT04644068), I/IIa EvoPAR‐BR01 (NCT06380751), III EvoPAR‐PR02 (NCT06952803), III	
	AZD9574	By selectively binding to and “trapping” PARP1 on damaged DNA, AZD9574 prevents base excision repair (BER), leading to the accumulation of DNA strand breaks and genomic instability, ultimately triggering cancer cell death (apoptosis).	CERTIS1 (NCT05417594), I/IIa	
	Fluzoparib	Inhibiting PARP enzymes and induces DNA‐double strands breaks, G2/M arrest and apoptosis in homologous recombination repair‐deficient cells	BCTOP‐L‐A01 (NCT05891093), III NCT07162051, II NCT05834582, II NCT05656131, II NCT06561022, II NCT06254066, II IMPARP (NCT05761470), II NCT06255392, III	
PARP1/2 + Tankyrase 1/2	Stenoparib (E7449/2X‐121)	Working through a dual‐action mechanism, inhibiting both PARP to disrupt DNA repair in tumor cells and tankyrase enzymes to interfere with the Wnt/β‐catenin signaling pathway		
Class I PI3K + mTORC1/2	Gedatolisib (PF‐05212384)	A pan‐class I PI3K and mTOR inhibitor that works by binding to and inhibiting the PI3K and mTOR kinases, which are involved in cell growth, proliferation, and survival pathways	NCT03911973, II VIKTORIA‐1 (NCT05501886), III VIKTORIA‐2 (NCT06757634), III	PI3K/AKT/mTOR Inhibitors: 11/32
Class I PI3K	MEN1611	Inhibiting the p110α isoform of PI3K, inducing a dose‐dependent depletion of the p110α protein, and modulating the tumor microenvironment by promoting a proinflammatory macrophage phenotype	SABINA (NCT05810870), II	
PI3Kα	STX‐478	Binding to a distinct allosteric site on the mutant enzyme, preventing its function and leading to tumor regression without causing the severe metabolic side effects (like hyperglycemia) associated with earlier, nonselective PI3Kα inhibitors	NCT05768139, I/II	
PI3Kδ/γ	Tenalisib (RP6530)	Inhibiting the PI3K delta and gamma isoforms and prevents the activation of the PI3K/AKT‐mediated signaling pathway; this may lead to a reduction in cellular proliferation in PI3K delta/gamma‐expressing tumor cells. In addition, this agent modulates inflammatory responses through various mechanisms.	NCT06189209, II	
AKT1/2/3	Ipatasertib (GDC‐0068)	Binding to and inhibits the activity of Akt in a non‐ATP‐competitive manner, which may result in the inhibition of the PI3K/Akt signaling pathway and tumor cell proliferation and the induction of tumor cell apoptosis	TAKTIC (NCT03959891), I NCT05554380, II NCT04920708, II BARBICAN (NCT05498896), II FINER (NCT04650581), III	
TROP‐2	SKB264	Delivering a cytotoxic payload, a belotecan‐derived topoisomerase inhibitor, directly to cancer cells, inhibiting tumor growth and proliferation; upon cellular uptake, the linker releases the toxin, which then exerts its cytotoxic effect.	MK‐2870‐001 (NCT04152499), I/II NCT07054242, II NeoSaciA (NCT07109284), II SHINE‐HER (NCT07182721), II NCT07071337, III NCT05347134, III NCT06081959, III NCT06279364, III	ADCs (non‐HER2‐targeted): 16/20
HER‐3	Patritumab deruxtecan (HER3‐DXd)	Binding to HER3‐expressing tumor cells, delivering a topoisomerase I inhibitor payload that induces targeted cell death	MK‐1022‐009 (NCT06686394), Ib/II ICARUSBREAST02 (NCT06298084), Ib/II NCT04965766, II NCT06172478, II MK‐1022‐010 (NCT06797635), II VALENTINE (NCT05569811), II TUXEDO‐3 (NCT05865990), II MK‐1022‐016 (NCT07060807), III	
PD‐1	Pucotenlimab (HX008)	Binding to PD‐1 and blocks its interaction with its ligands, PD‐L1 and PD‐L2, thereby restoring the ability of immune cells to target cancer cells	NCT06889610, II	ICIs: 69/170
	Dostarlimab (TSR‐042)	An anti‐PD‐1 monoclonal antibody that binds with high affinity to the PD‐1 receptor and effectively blocks interaction with PD‐L1 and PD‐L2, restoring cytotoxic T‐cell activity and freeing the T‐cell to kill tumour cells	NCT04673448, Ib NCT04584255, II NCT04837209, II	
	Cemiplimab (REGN2810)	Blocking the PD‐1 receptor, preventing binding and activation of PD‐L1 and PD‐L2; the inhibition of the PD‐1 pathway results in immune checkpoint enhancing T‐cell‐mediated immune response leading to T‐cell activation and proliferation against tumor cells.	NCT05064085, I NCT06860815, II CemiHALT (NCT04243616), II	
	Sintilimab	A fully human IgG4 monoclonal antibody that binds to programmed cell death receptor‐1 (PD‐1), thereby blocking the interaction of PD‐1 with its ligands (PD‐L1 and PL‐L2) and consequently helping to restore the endogenous antitumor T‐cell response.	NCT06308939, II NeoSACT (NCT04877821), II NeoSTEP (NCT05843292), IV	
	Camrelizumab	Blocking the PD‐1 pathway, which allows tumors to evade the immune system; as a humanized monoclonal antibody, it binds to PD‐1 receptors on immune cells, preventing tumor cells from interacting with their PD‐L1 ligands.	NCT04395989, II NCT06313463, III NCT05760378, III NCT06889688, III BCTOP‐T‐N01 (NCT05999149), III … A total of 28 trials	
	Nivolumab	Binding to and blocking the protein PD‐1 on the surface of some cancer cells, which keeps cancer cells from suppressing the immune system; this allows the immune system to attack the cancer cells.	NCT03650894, II NCT03789110, II NCT03414684, II … A total of 27 trials	
CTLA‐4	Tremelimumab	An anti‐CTLA4 monoclonal antibody that blocks the inhibitory signal resulting from CTLA‐4 binding to CD80/86, leading to prolongation and enhancement of T‐cell activation and expansion.		
TIGIT	Tiragolumab	Targeting T‐cell immunoreceptor on activated T‐cell and NK cell subsets and prevents interaction with CD155 (poliovirus receptor) to limit cellular proliferation, effector cytokine production, and killing of target tumor cells.	SKYLINE (NCT06175390), II TONIC‐3 (NCT06342037), II	
	Domvanalimab	An Fc‐silent humanized IgG1 anti‐TIGIT monoclonal antibody that blocks interaction between TIGIT and its ligand CD155, thus reducing immunosuppression of T‐cells and NK cells, promoting antitumor activity.	NCT07134556, II	
LAG‐3	Relatlimab	Blocking the lymphocyte‐activation gene 3 (LAG‐3) pathway; by binding to and inhibiting the LAG‐3 receptor on T cells, relatlimab prevents LAG‐3 from interacting with its ligands.	SIMONE (NCT06963905), Ib	
GRPR	Lu‐NeoB ([177Lu]Lu‐NeoB/177Lu‐NeoBOMB1)	Targeting and binding to GRPRs present on certain tumor cells; upon binding and internalization, this radioconjugate specifically delivers a cytotoxic dose of beta radiation to GRPR‐expressing cells.	NCT05870579, I NeoB‐Cap1 (NCT06247995), I/II	innovative technology: 2/2

All trials in the table were retrieved from ClinicalTrials.gov (https://clinicaltrials.gov/, accessed on September 20, 2025).

*Abbreviations*: estrogen receptor (ER); selective estrogen receptor modulators (SERMs); selective estrogen receptor degraders (SERDs); proteolysis‐targeting chimera (PROTAC); complete estrogen receptor antagonist (CERAN); selective estrogen receptor covalent antagonist (SERCA); ovarian function suppression (OFS); human epidermal growth factor receptor 2 (HER2); antibody–drug conjugates (ADCs); tyrosine kinase inhibitor (TKI); cyclin‐dependent kinase (CDK); poly (ADP‐ribose) polymerase (PARP); phosphatidylinositol 3‐kinase–protein kinase B–mechanistic target of rapamycin signaling pathway (PI3K–AKT–MTOR); human epidermal growth factor receptor 3 (HER3); programmed cell death protein 1 (PD‐1); programmed death‐ligand 1/2 (PD‐L1/2); immune checkpoint inhibitors (ICIs); cytotoxic T‐lymphocyte antigen 4 (CTLA‐4); T cell immunoreceptor with Ig and ITIM domains (TIGIT); lymphocyte activation gene‐3 (LAG‐3); gastrin‐releasing peptide receptor (GRPR).

### 3.2 HER2‐Targeted Therapies

The identification of HER2 (also known as ERBB2) marked a major advance in breast cancer therapeutics. Targeted strategies, including monoclonal antibodies, tyrosine kinase inhibitors (TKIs), and ADCs, have transformed outcomes for patients with HER2(+) disease. Trastuzumab is the first humanized monoclonal antibody targeting HER2. The research on trastuzumab has expanded from the initial metastatic breast cancer to the neoadjuvant and adjuvant treatment of early breast cancer [113, 114]. Treating with trastuzumab is associated with cardiac toxicity, necessitating monitoring of left ventricular ejection fraction [115]. However, many patients ultimately experience resistance and relapse [116].

The development of pertuzumab and its combination with trastuzumab significantly improved treatment efficacy [116]. The Phase III clinical trial of CLEOPATRA showed that, compared with trastuzumab + docetaxel, the treatment of HER2(+) metastatic breast cancer with pertuzumab combined with trastuzumab and docetaxel significantly prolonged the median PFS and OS of patients with HER2(+) metastatic breast cancer [117, 118]. Yet, in early breast cancer, the APHINITY trial showed only a modest absolute benefit in invasive DFS (iDFS; 4.9% improvement in node‐positive patients; HR 0.72, 95% CI 0.60–0.87) without OS advantage, demonstrating the challenges in achieving durable benefit in the curative setting [119].

Beyond trastuzumab and pertuzumab, other HER2‐directed antibodies are under investigation. In the Phase III SOPHIA trial, margetuximab plus chemotherapy modestly improved PFS compared with trastuzumab‐based therapy [120]. Further exploration analyses suggest differential efficacy by CD16A genotype [121]. These findings highlight the potential importance of host immunogenetic factors in optimizing antibody therapy.

Small‐molecule TKIs have further expanded therapeutic options. Lapatinib is an oral TKI that can dual inhibit EGFR (HER1) and HER2 to exert antitumor activity [116]. Lapatinib has unique advantages in the treatment of recurrent HER2(+) breast cancer because it can pass through the blood‐brain barrier, where up to 50% of patients with HER2(+) BC develop brain metastases [116]. The Phase II study LANDSCAPE results support the combination of lapatinib and capecitabine as a first‐line treatment for brain metastasis of HER2(+) breast cancer [122]. Neratinib, an irreversible pan‐HER TKI, has shown sustained activity in HER2‐mutated and amplified tumors [123]. The Phase III NALA trial demonstrated that, similar to lapatinib, the combination of naratinib and capecitabine improved the prognosis of HER2(+) metastatic breast cancer [124]. More recently, the HER2‐selective TKI tucatinib has demonstrated superior activity with favorable tolerability. In the HER2CLIMB trial, the addition of tucatinib to capecitabine and trastuzumab significantly prolonged PFS (7.8 vs. 5.6 months, HR 0.54) and OS (21.9 vs. 17.4 months, HR 0.66), including in patients with brain metastases, establishing this regimen as a new standard [125]. Ongoing basket trials are assessing tucatinib activity in HER2‐mutant cancers beyond breast cancer (Table 1).

ADCs have greatly promoted the treatment of HER2(+) breast cancer. Ado‐trastuzumab emtansine (T‐DM1) is the first developed anti‐HER2 ADC. It improved both PFS (9.6 vs. 6.4 months, HR 0.65) and OS (30.9 vs. 25.1 months, HR 0.68) in the EMILIA trial, compared with lapatinib and capecitabine [126]. In the KAMILLA trial, T‐DM1 also showed its effectiveness in a subgroup of brain metastasis patients. Another ADC, T‐DXd, has demonstrated superior potency and broader applicability. In the DESTINY–Breast03 trial, T‐DXd significantly improved outcomes in patients previously treated with trastuzumab and T‐DM1, with a response rate of 60.9% [127]. DESTINY–Breast12 prospective study on T‐DXd shows significant and persistent intracranial activity of T‐DXd [128]. Importantly, the DESTINY–Breast06 study extended these benefits to patients with HER2‐low or ultralow tumors (median PFS 13.2 vs. 8.1 months, HR 0.62), leading to regulatory approval and redefining the biological and therapeutic boundaries of HER2 [129]. The results also greatly promoted the therapeutic advances to the HER2‐low subtype. The safety and efficacy of T‐DXd remain to be fully elucidated. As interstitial lung disease remains a safety concern, careful monitoring is needed. Ongoing trials such as NCT06551220, NCT06429761, NCT06210776, and NCT07030569 may provide valuable insights.

Disitamab vedotin is the first next‐generation agent that is expanding therapeutic options. This agent combines direct cytotoxicity with immune‐mediated activity and has shown promising efficacy in both HER2(+) and HER2(−) advanced breast cancer, with objective response rates (ORR) of 42.9 and 33.3%, respectively [130]. Phase III studies such as ROSY (NCT05904964) and NCT06278870 are underway to clarify its role.

Taken together, HER2‐directed therapies now span monoclonal antibodies, TKIs, and ADCs, achieving substantial improvements in survival for many patients. However, resistance and relapse are common, durable benefit in early‐stage disease is uncertain, and safety profiles require vigilance. Future advantages may rely on integrating these agents, refining patient selection through molecular and immunogenetic markers, and extending benefits to HER2‐low and HER2‐mutant subsets.

### 3.3 Targeting Cell Cycle and DNA Repair

Cell cycle dysregulation is a central mechanism of tumorigenesis. CDK4/6i inhibit the proliferation of malignant cells by binding to the ATP domain of CDK4/6, which plays a key role in the G1‐S checkpoint [67], and then inducing G1 arrest [131]. When combined with endocrine therapy, CDK4/6i are widely used as first‐ or second‐line treatment for patients with hormone receptor‐positive, HER2(−) advanced breast cancer.

The PALOMA‐2 trial confirmed that compared with placebo plus letrozole in ER(+)/HER2(−) advanced breast cancer, the PFS of palbociclib plus letrozole was statistically and clinically significantly improved [132]. However, extended follow‐up showed no statistically significant OS benefit (53.8 vs. 49.8 months; HR = 0.92) [133]. This contrasts with the MONALEESA‐2 trial, where ribociclib plus letrozole conferred a clear OS advantage of more than 12 months [134]. Similarly, abemaciclib in MONARCH‐3 improved median OS when combined with nonsteroidal AIs [135]. Dalpiciclib also demonstrated substantial PFS benefit in the DAWNA‐2 trial (30.6 vs. 18.2 months; HR = 0.51), suggesting its potential as a new first‐line standard in hormone receptor(+)/HER2(−) advanced breast cancer [136]. By contrast, the SONIA study raised important questions about optimal treatment sequencing, showing no significant benefit of CDK4/6i in the first‐line setting compared with later introduction, but with increased toxicity due to longer exposure [137]. These results emphasize that while CDK4/6i are effective, however, the optimal timing of CDK4/6i introduction warrants careful deliberation. Moreover, resistance invariably develops, and predictive biomarkers remain limited. Aside from ER positivity, which guides clinical use, candidate markers such as cyclin D, CDKN2A, and RB1 status have yet to be translated into practice [137].

PARP inhibitors are the first targeted therapy that can improve the prognosis of patients with hereditary tumors [138]. PARP inhibitors (PARPi) have two main mechanisms of action: (1) Catalytic inhibition through competition with NAD+ binding. (2) Generation of PARP–DNA complexes that block replication forks [139]. Cancer patients with *BRCA1* or *BRCA2* germline mutations are sensitive to PARPi [140]. Olaparib is the first developed PARPi, which have been proved by the OlympiA trial to improve OS in germline BRCA1/2‐mutated early breast cancer (HR = 0.68) [141]. The latest trial of neoadjuvant olaparib with chemotherapy in TNBC showed that in patients with germline *BRCA1* and *BRCA2* wild‐type TNBC, neoadjuvant olaparib combined with carboplatin‐paclitaxel and anthracycline chemotherapy did not improve the pathologic complete response (pCR) rate, event‐free survival (EFS) or OS [142].

Other agents have shown efficacy in broader contexts. Talazoparib demonstrated benefit in germline PALB2‐mutated advanced breast cancer [143, 144]. Veliparib improved PFS in BRCA‐mutated or “BRCA‐like” TNBC when added to chemotherapy (BROCADE3, HR = 0.71) [145]. New agent AZD5305, which is a selective PARP1 inhibitor, aims to minimize hematologic toxicity associated with dual PARP1/2 blockade [146]. However, trials of PARPi in *BRCA* wild‐type disease, including neoadjuvant settings, have largely been negative, suggests the need for more precise patient selection. Resistance to PARPi occurs in nearly all patients. Although several resistance mechanisms have been identified, how to effectively target them is largely unknown. Combining PARP inhibitors with ADCs may be a potential solution [141, 147].

### 3.4 PI3K/AKT/mTOR Inhibitors

Dysregulation of the PI3K/AKT/mTOR pathway is among the most frequent molecular alterations in breast cancer and plays a central role in resistance to both endocrine therapy and CDK4/6 inhibition [148]. PI3K/AKT/mTOR inhibitors targeting this pathway have a positive impact on strengthening the treatment and improving the prognosis of breast cancer patients.

Alpelisib is the first oral α‐selective PI3K inhibitor. SOLAR‐1 trial showed that fulvestrant plus alpelisib had statistically significant and PFS benefits on PIK3CA‐mutation, hormone receptor‐positive, HER2(−) advanced breast cancer [39, 149]. Although the numerical improvement in median OS in the follow‐up analysis was 7.9 months, it did not cross the predetermined statistical significance boundary [150] [median OS was 39.3 months (95% CI 34.1–44.9) for alpelisib–fulvestrant and 31.4 months (95% CI 26.8–41.3) for placebo‐fulvestrant (HR = 0.86, 95% CI 0.64–1.15; *p* = 0.15)]. Subsequent trials of alpelisib monotherapy confirmed activity in ER(+) disease but not in TNBC [151]. Inavolisib is a novel potent PI3Kα‐selective inhibitor. In a Phase III, double‐blind, randomized trial, INAVO120, inavolisib plus palbociclib–fulvestrant significantly prolonged both PFS (15.0 vs. 7.3 months) [152] and OS (34.0 vs. 27.0 months) [153] compared with placebo. It has now been approved by the US FDA and NMPA for endocrine‐resistant, *PIK3CA* mutated, hormone receptor‐positive, HER2(−) locally advanced or metastatic breast cancer. Nevertheless, adverse events remain a limiting factor, with discontinuation rates notably higher than placebo. Efforts to improve safety have led to the development of more selective agents, such as STX‐478 [154] and tenalisib, which are currently in early‐phase trials and may offer a more favorable therapeutic index.

AKT inhibitors have also shown promise for downstream targets in the pathway. Capivasertib selectively inhibits all three Akt subtypes. It improved both PFS and OS in the Phase II FAKTION trial [155] and demonstrated consistent PFS benefit in the Phase III CAPItello‐291 study [156], including in patients previously treated with CDK4/6i. These findings have been reinforced in the expanded Chinese cohort, suggesting broad clinical relevance [157]. By contrast, ipatasertib failed to improve outcomes in either the neoadjuvant FAIRLANE trial [158] or the Phase III IPATunity130 study [159], highlighting the variability of efficacy within AKT inhibitors. Resistance mechanisms and toxicity remain critical challenges. Novel agents such as the dual p70S6K/AKT inhibitor M2698 have shown manageable safety and preliminary antitumor activity in resistant disease [160].

Targeting mTOR has been explored with everolimus, an allosteric inhibitor of mTORC1. The BOLERO‐2 trial established everolimus plus exemestane as an effective strategy for endocrine‐resistant, hormone receptor–positive/HER2(–) advanced breast cancer, leading to regulatory approval [161]. A recent Phase III randomized, placebo‐controlled trial evaluated the efficacy of everolimus + endocrine therapy as adjuvant therapy for high‐risk, hormone receptor‐positive/HER2(−) breast cancer after adjuvant/neoadjuvant chemotherapy [162]. The results showed that 1 year of everolimus + endocrine therapy adjuvant therapy did not improve the overall results [162]. Gedatolisib is a dual inhibitor of class I PI3K and mTOR. In an open‐label Phase Ib trial, gedatolisib plus palbociclib and endocrine therapy showed a promising objective response rate (ORR) with acceptable safety in women with hormone receptor‐positive, HER2(−) advanced breast cancer [163].

Overall, inhibitors of the PI3K/AKT/mTOR axis have expanded treatment options for patients with hormone receptor‐positive breast cancer, particularly those with PIK3CA mutations. Yet, the clinical impact is tempered by issues of drug resistance, limited efficacy in TNBC, and treatment‐limiting toxicities. Ongoing trials of next‐generation inhibitors with improved selectivity will be crucial in defining whether these limitations can be overcome and whether broader patient populations can benefit from pathway‐targeted strategies.

### 3.5 ADCs for Novel Targets

ADCs are rapidly reshaping the therapeutic landscape of breast cancer. Sacituzumab govitecan (SG), the first‐in‐class Trop‐2 ADC, significantly improved PFS (5.5 vs. 4.0 months; HR = 0.66) and OS (14.4 vs. 11.2 months; HR = 0.79) comparing with chemotherapy in the TROPiCS‐02 trial, with consistent benefits reported in Asian populations. Hematologic toxicities such as neutropenia and anemia remain the most frequent high‐grade adverse events [164, 165, 166, 167]. Datopotamab deruxtecan (Dato‐DXd) is a trop2 ADC with a humanized IgG1 mAb conjugated to a Top1 inhibitor [165]. The results from the Phase I TROPION‐PanTumor01 study showed that Dato‐DXD showed promising clinical activity in patients with advanced hormone receptor‐positive/HER2(−) BC and TNBC [168]. The ORRs of hormone receptor‐positive/HER2(−) BC and TNBC patients were 26.8% (95% CI 14.2–42.9) and 31.8% (95% CI 18.6–47.6), respectively [168]. Phase III trials, such as the tropion‐breast01 and tropion‐breast02 trial, are conducting further evaluation and research on Dato‐DXD.

Sacituzumab tirumotecan has been approved by the China National Drug Administration for the treatment of adult patients with unresectable locally advanced or metastatic TNBC who have received at least two systemic therapies. The Phase III OptiTROP‐Breast01 trial evaluated the therapeutic efficacy of Sacituzumab tirumotecan in patients with locally recurrent or metastatic TNBC [169]. The results showed that Sacituzumab tirumotecan had a statistically significant and clinically significant improvement in PFS compared with chemotherapy (median 6.7 vs. 2.5 months, HR = 0.32; 95% CI 0.24–0.44; *p* < 0.00001) [169]. Although some targeted ADCs have shown clinical benefits and have been used as standard treatments, clinical needs remain unmet due to accessibility differences. The application of new ADCs in breast cancer still has great prospects. Patritumab deruxtecan (HER3 DXD) may be one of them [170].

Although ADCs have yielded impressive responses, not all trials have met expectations. Pertuzumab‐containing regimens in the APHINITY trial improved invasive DFS but failed to show a clear OS benefit at interim analyses [119]. Similarly, some ADCs demonstrate high response rates without durable benefit, and toxicity such as interstitial lung disease with T‐DXd remains a concern [171]. These findings caution against overgeneralizing early positive signals and highlight the need for long‐term follow‐up and real‐world validation.

### 3.6 Immunotherapy and Combination Approaches

Immunotherapy controls and eliminates tumors by mobilizing the host immune system, offering new therapeutic opportunities for breast cancer subtypes lacking clear treatment targets, such as TNBC. Among the most widely investigated approaches are immune checkpoint inhibitors (ICIs), evaluated both as monotherapy and in combination with chemotherapy or targeted therapy.

Pembrolizumab is a monoclonal antibody that binds to the PD‐1 receptor and blocks its interaction with PD‐L1 and PD‐L2, releasing PD‐1 pathway‐mediated inhibition of the immune response, including the antitumor immune response. In the Phase III KEYNOTE‐119 trial, its superiority over chemotherapy was evident only in metastatic TNBC patients with high PD‐L1 expression [172]. More robust evidence came from KEYNOTE‐355, where pembrolizumab plus chemotherapy significantly prolonged PFS (9.7 vs. 5.6 months; HR = 0.65, 95% CI 0.49–0.86) and OS (23.0 vs. 16.1 months; HR = 0.73, 95% CI 0.55–0.95) in PD‐L1–positive (CPS ≥ 10) metastatic TNBC [173, 174]. In the early disease setting, KEYNOTE‐522 confirmed that adding pembrolizumab to neoadjuvant chemotherapy, followed by adjuvant pembrolizumab, improved both EFS (84.5 vs. 76.8% at 36 months) and OS (86.6 vs. 81.7% at 60 months) [175, 176]. Notably, exploratory analyses suggested benefit even among patients who did not achieve a pCR, underscoring a broader role beyond pCR improvement [177, 178].

The efficacy of pabolizumab in non‐TNBC is being explored. Encouraged by these results, ongoing trials are assessing pembrolizumab in non‐TNBC subtypes. KEYNOTE‐756 showed a significant increase in pCR (24.3 vs. 15.6%; *p* = 0.00005) when pembrolizumab was added to neoadjuvant chemotherapy in high‐risk, early‐stage ER(+)/HER2(−) breast cancer, although EFS data remain immature [179]. Preliminary findings from the Phase II WSG‐KEYRICHED‐1 study also point to therapeutic potential when pembrolizumab is combined with dual HER2 blockade in HER2‐enriched early breast cancer [180].

Other ICIs have produced mixed outcomes. Atezolizumab is a monoclonal antibody that binds to PD‐L1 and blocks its interactions with both PD‐1 and B7.1 receptors. In the IMpassion130 trial, atezolizumab combined with nanoparticle albumin‐bound‐paclitaxel (nab‐paclitaxel) prolonged PFS in the treatment of mTNBC [181]. However, the results of IMpassion130, a Phase III randomized clinical trial, showed that compared with paclitaxel alone, the combination of atezolizumab and paclitaxel did not improve PFS and OS in patients with advanced TNBC [182]. Therefore, the US FDA withdrew the previously approved alitizumab for TNBC patients with PD‐L1 expression. Tislelizumab, with enhanced PD‐1 binding affinity, achieved a pCR rate of 68% in the Phase II TREND trial when combined with anthracycline‐ and taxane‐based chemotherapy in TNBC [183]. Moreover, it has shown improved PFS in PD‐L1‐positive advanced TNBC in the Phase III TORCHLIGHT trial [184]. nivolumab significantly increased pCR rates in high‐risk ER+/HER2– disease in CheckMate 7FL, particularly among PD‐L1–high patients [185].

Although ICIs have been studied rapidly in breast cancer, their therapeutic effect is limited by tumor heterogeneity, tumor subtypes and TME. The identification of prognostic biomarkers other than PD‐L1 is helpful to enhance the efficacy of ICIS. In addition, the development of adoptive T cell immunotherapy and anticancer vaccines are also further explored in the immunotherapy of breast cancer.

### 3.7 Mechanisms of Resistance and Overcoming Therapeutic Failure

Drug resistance is a problem that cannot be ignored in drug therapy. Although most ER(+) tumors are initially sensitive to anti estrogen drugs, they will develop resistance with the extension of antiestrogen drug treatment time. It is usually characterized by clinical recurrence and involves genetic and epigenetic changes. Tumors that initially rely on estrogen for proliferation usually evolve to reactivate ER‐directed signals under anti estrogen treatment. This points out the direction for the development of next‐generation endoplasmic reticulum targeted therapies. LBD acquired mutations in *ESR1* are important drivers of treatment resistance [186, 187, 188, 189, 190]. It is the main source of AIs resistance [191, 192]. In addition to *ESR1* point mutations, structural variations of *ESR1* are also involved in therapeutic resistance, such as *ESR1* fusion [193]. HER2‐amplified tumors usually show therapeutic resistance to endocrine therapy at the time of initial drug exposure [193]. *ERBB2*‐point mutation is an acquired drug resistance mechanism. *ERBB2*‐mutated tumors have reduced sensitivity to estrogen inhibitors, SERM tamoxifen, SERD fulvestin, and CDK4/CDK6 inhibitors. The activation of PI3K–Akt pathway and RAS–RAF–MAPK pathway (RTK downstream signaling pathway) can induce breast cancer cells to resist endocrine therapy. Evidence shows that the activation of RTK and its downstream signaling effectors promotes the proliferation of ER(+) breast cancer cells independent of ER activity, and the alteration of transcription factors other than ER is considered as a potential mechanism of endocrine resistance. Amplification and hotspot mutations of MYC and CTCF are common in tumors that progress after metastatic endocrine therapy [192]. Mutations in FOXA1 are also strongly associated with the prognosis of ER(+) breast cancer patients treated with AIs [194]. Approximately 10–20% of metastatic ER(+) breast tumors exhibit loss or reduction of ER expression [195]. This suggests that lineage plasticity may also play a role in breast cancer treatment resistance. In breast cancer treated with endocrine therapy, a large part of endocrine therapy resistance is related to the characteristics and limitations of endocrine therapy nowadays, so one of the effective means to solve endocrine therapy resistance is to develop more optimized ER‐targeted drugs. Oral SERDs, such as elacestrant, have demonstrated efficacy in *ESR1*‐mutant tumors, suggesting that it can be a common mechanism of endocrine resistance. In the EMERALD Phase III trial, elacestrant significantly improved PFS compared with standard endocrine therapy in patients with *ESR1* mutations [196] (HR = 0.55; 95% CI: 0.39–0.77; *p* = 0.0005). These agents not only degrade ER but also modulate immune‐related gene expression, potentially enhancing tumor immunogenicity.

Other SERDs, such as camizestrat and giredstrat, have also shown clinical benefits [197, 198], but further Phase III clinical trials are still ongoing. The new endocrine therapy proteolysis‐targeting chimaera (PROTAC) and selective ER covalent antagonists (SERCAs) have expanded the understanding of the path of endocrine therapy. Emerging evidence indicates that both PROTAC technology and next‐generation SERCAs can restore drug sensitivity in endocrine‐resistant, hormone receptor‐positive tumors by rectifying the tumor–immune interface [199]. CDK4/6i, including palbociclib and abemaciclib, induce cell cycle arrest and promote T‐cell activation by suppressing Tregs and increasing antigen presentation. In preclinical models, CDK4/6i upregulated MHC class I expression and enhanced PD‐L1 sensitivity, suggesting synergy with ICIs [200]. Inhibition of CDK4/6 combined with ER inhibition is currently the most effective combination treatment strategy in clinic. In addition, PI3K‐Akt‐mTOR pathway activation, which is commonly seen in ER(+) patients, is also associated with endocrine resistance. Drugs targeting the PI3K–Akt–mTOR pathway, such as alpelisib, have been shown to have significant PFS improvements [39]. The emergence of ADCs has changed the traditional treatment. ADCs, such as T‐DXd, offer targeted cytotoxicity with immune‐stimulatory bystander effects. They may overcome immune resistance by inducing immunogenic cell death and enhancing tumor‐infiltrating lymphocyte activity. These drugs can not only control the cell cycle while being well tolerated but also take into the account for the tumor heterogeneity between and within patients.

TME is crucial in immunotherapy. Stromal cells such as CAFs, TAMs, and MDSCs are all associated with immune resistance. CAFs, TAMs, and MDSCs induce the differentiation of Treg cells, antigen uptake and maturation of dendritic cells, and M2 polarization, which also inhibit the activation and recruitment of CD8+ and NK cells [79]. Tumor metabolism, especially glycolysis and TCA cycle, is also associated with immune tolerance in breast cancer [79]. Targeting these cells and their metabolic regulation, combined therapy can overcome immune resistance and improve the efficacy of breast cancer immunotherapy. In addition, new therapeutic methods such as photodynamic therapy, photoimmunotherapy, and nanoparticle mediated drug delivery are being explored [201, 202, 203].

### 3.8 Emerging Targets and Future Directions

In addition to established therapies, several novel targeted agents are under clinical or preclinical investigation (Table 1). Vepdegestrat is an oral PROTAC ER degrader that can function using the ubiquitin proteasome system [204]. In the Phase III VERITAC‐2 trial for advanced breast cancer, vepdegestrat significantly prolonged PFS in patients harboring ESR1 mutations compared with fulvestrant (5.0 vs. 2.1 months; HR = 0.58; 95% CI 0.43–0.78; *p* < 0.001) [205]. Palazestrant is a novel oral complete ER antagonist and SERD. Palazestrant can completely block estrogen‐induced transcriptional activity. Palazestrat showed potent antitumor activity in preclinical xenograft models of both *ESR1* wild‐type and mutant human breast cancer [206]. A Phase I/Ⅱ study in heavily pretreated ER(+)/HER2(−) advanced or metastatic breast cancer patients confirmed its clinical efficacy in both wild‐type and ESR1‐mutant tumors [207]. Two Phase III studies of palazestrant OPERA‐01 (NCT06016738) and OPERA‐02 (NCT07085767) are ongoing.

Personalized therapy based on molecular profiling has emerged as a key research direction in targeted therapy. Oncotype DX was the first genomic biomarker test for breast cancer treatment. Oncotype DX was the first genomic assay developed to assess recurrence risk in ER‐positive, HER2‐negative, lymph node–negative invasive breast cancer and to guide tamoxifen therapy duration [208]. MammaPrint contains a 70‐gene signature, provides prognostic insight and can identify low‐risk, nonmetastatic tumors [208]. PAM50 detection provides additional data on tumor biology and quantitative information on proliferation, intracavitary gene expression, *ESR1*, *PGR*, and *ERBB2* [208]. In addition, molecular profiling tests such as EndoPredict and Breast Cancer Index have been applied in clinical practice [208]. Dynamic circulating tumor DNA (ctDNA) monitoring has great benefits for personalized diagnosis and treatment. Some trials [209, 210, 211] establish ctDNA as a versatile and clinically actionable biomarker across the breast cancer care pathway: first, in early‐stage disease, ctDNA enables response‐guided therapy and early relapse detection. Next, in advanced disease, it facilitates real‐time genomic profiling and precision therapy selection.

Dynamic ctDNA monitoring offers significant advantages for personalized management. A ctDNA sequencing platform based on whole‐genome sequencing demonstrates higher ctDNA detection rates in early‐stage breast cancer than tumor‐informed assays based on exomes, representing innovation and advancement in sequencing‐platform technology [212]. With continued validation, ctDNA is poised to become a cornerstone of precision oncology in breast cancer, supporting personalized, dynamic, and evidence‐based treatment strategies.

scRNA‐seq provides high‐resolution transcriptomic analysis, elucidating tumor heterogeneity and informing targeted and individualized therapies [92]. Molecular subtypes and stages, genetic markers, and genetic changes of breast cancer all have an impact on the treatment scheme. Individualized and precise breast cancer treatment needs to be continuously promoted.

## 4 Risk Assessment and Stratification

Accurate risk assessment and stratification form the cornerstone of breast cancer prevention, early detection, and personalized management. Traditional models based on family history, reproductive factors, and lifestyle exposures provide useful but limited estimates of individual risk (Table 2). The advances in genomics, imaging, and computational analytics open a way to refine prediction, guide tailored surveillance or preventive interventions through integrating germline mutations, PRS, breast density, and emerging biomarkers. In this section, we summarize the evolution of risk assessment strategies, highlight their strengths and limitations, and discuss how integrating novel tools with established models may optimize clinical decision‐making and resource allocation in diverse populations.

**TABLE 2:** Summary of breast cancer screening recommendations in high‐risk women.

Organization	Published year	Country/region	References	Definitions of high‐risk population	Age range for screening	Screening methods for different age group	Screening intervals
NCCN	2025	United States	[213]	Individuals with confirmed or suspected BRCA1/2 pathogenic/likely pathogenic germline variants based on testing and/or family history	25–29	MRI; MAM (only if MRI unavailable or individualized if family BC <30 years)	Annual
					30–75	MAM + MRI	Annual
CACA	2024	China	[214]	(1) Individuals with a significant hereditary predisposition to breast cancer can be identified based on the following criteria:	Starting <40	MAM ± MRI	Annual
				① A first‐degree relative has a history of breast cancer or ovarian cancer			
				② Two or more second‐degree relatives diagnosed with breast cancer before the age of 50 years			
				③ Two or more second‐degree relatives diagnosed with ovarian cancer before the age of 50 years			
				④ At least one first‐degree relative carries a known pathogenic mutation in the BRCA1/2 genes, or the individual themselves carries a pathogenic mutation in the BRCA1/2 genes			
				(2) Individuals with a prior diagnosis of atypical ductal or lobular hyperplasia, or lobular carcinoma in situ (LCIS)			
				(3) Individuals who received chest radiation therapy before the age of 30 years		US, CBE	Every 6–12 months
				(4) Using the Gail model to assess breast cancer risk based on multiple risk factors, including the individual's age, ethnicity, age at menarche, age at first childbirth, personal history of breast disease, family history of breast cancer, and number of breast biopsies. If the estimated risk of developing breast cancer within the next 5 years is ≥1.67%, the individual is considered to be at high risk.		MRI, when necessary	NA
EMSO (Pan‐Asian)	2024	China, Indonesia, India, Japan, Korea, Malaysia, Philippines, Singapore, Taiwan, and Thailand	[215, 216]	Women with a strong family history or known germline BRCA1/2 mutations (gBRCA1/2m) and other high‐risk pathogenic variants (PVs)	Start at the age of 30 years, or 5 years younger than the youngest family member with breast cancer	MRI+(may be considered in between annual MRI studies) ①30–39: US ± MAM; ②≥40: MAM ± US	MAM: annual(but every 6 month for BRCA1); ①and②:may be considered in between annual MRI studies
EMSO	2023	Europe	[215]	Women with a strong family history or known germline BRCA1/2 mutations (gBRCA1/2m) and other high‐risk pathogenic variants (PVs)	Start at the age of 30 years, or 5 years younger than the youngest family member with breast cancer	MRI+(may be considered in between annual MRI studies) ①30–39: US ± MAM; ②≥40: MAM ± US	MAM: annual(but every 6 month for BRCA1); ①and②:may be considered in between annual MRI studies
ACR	2023	United States	[217]	Women with genetics‐based increased risk (and their first‐degree untested relatives) or with a calculated lifetime risk of 20% or more	Starting at 30	DM, ±DBT; MAM	DM, ± DBT: NA; MAM: annual
				BRCA carriers	Starting at 30	MRI, MAM	Annual
					Starting at 25	MRI; and MAM starting at 40	Annual
BH	2023	Bosnia and Herzegovina (BiH)	[218]	High risk is defined as a >20% lifelong risk by risk modeling (Tyrer‐Cuzick model)	NA	MRI and MAM, alternately	Alternately every 6 months
CBR, SBM, and FEBRASGO	2023	Brazilian	[219]	Women with a pathogenic mutation of the BRCA1 gene or not tested, but with first‐degree relatives who are carriers, or a strong family history of breast cancer (lifetime risk ≥ 20%)	BRCA1: ≥35; other: 30	MRI: BRCA1: ≥25 and TP53: ≥20 and BRCA2: ≥30; MAM: BRCA1: ≥35 and other: ≥30; ±US	Annual
NCC	2020	China	[220]	Personal history of precancerous lesions and/or breast cancer	NA	MAM and US	Annual
				Family history of breast cancer	NA	MAM and US	Annual
				Known genetic predisposition of breast cancer	NA	MRI	Annual
				History of mantle or chest radiation therapy	NA	MRI	Annual
				Dense breasts	NA	MAM and US	Annual
ASCO	2020	United States	[221]	Men with a history of breast cancer and a genetic predisposing mutation	NA (male)	MAM	Annual

*Abbreviations*: National Comprehensive Cancer Network (NCCN); Chinese Anti‐Cancer Association (CACA); European Society of Medical Oncology (EMSO); American College of Radiology (ACR); Bosnia and Herzegovina (BH); the Brazilian College of Radiology and Diagnostic Imaging, Brazilian Society of Mastology and Brazilian Federation of Gynecology and Obstetrics Association (CBR‐SBM‐FEBRASGO); American Cancer Society (ACS); National Cancer Center (NCC); American Society of Clinical Oncology (ASCO).

### 4.1 Risk Factors

Women with a genetic predisposition face a markedly elevated breast cancer risk. *BRCA1* and *BRCA2* mutations are the most common mutations in breast cancer, conferring lifetime risks of approximately 72 and 69% by the age of 80 years, respectively. *BRCA1* mutation carriers typically develop cancer in their 30s–40s, while *BRCA2* carriers peak later, in their 40s–50s [14]. *TP53* mutations, though rare, are associated with Li–Fraumeni syndrome and very early onset breast cancer, often before the age of 30 years [222]. *PALB2* mutations also confer substantial risk, with carriers having a 35% probability of developing breast cancer by the age of 70 years [223]. These mutations are further linked to other malignancies, including ovarian and pancreatic cancer, necessitating tailored surveillance such as early MRI and mammography, and in some cases, risk‐reducing interventions [224, 225, 226, 227]. Understanding gene‐specific risks enables more precise counseling and management for high‐risk women.

Family history of cancer remains an important risk factor, reflecting both inherited susceptibility and shared environmental exposures. The risk rises with the number of first‐degree relatives affected [15].

Women with a personal history of breast cancer or premalignant lesions such as atypical ductal hyperplasia, lobular carcinoma in situ, or ductal carcinoma in situ (DCIS) are at significantly higher risk of invasive disease [228]. Atypical ductal hyperplasia confers a four‐ to fivefold increased risk, lobular carcinoma in situ an eight‐ to tenfold risk, and DCIS carries a long‐term risk of progression lasting decades [16].

Prior mantle or chest radiation, especially during childhood or adolescence, increases lifetime breast cancer risk up to eight‐fold, with cumulative incidence reaching 13–20% by age the of 40–45 years, comparable to *BRCA* mutation carriers [17, 229, 230]. MRI offers superior sensitivity for radiation‐induced cancers, and combined screening significantly reduces breast cancer mortality in this population. Cost‐effectiveness analyses also support intensive surveillance protocols [231, 232].

Breast density represents another major independent risk factor. Women with dense breasts face a four‐ to six‐fold increased risk compared with those with fatty breasts [18, 233]. This is attributed both to the greater amount of glandular and stromal tissue and to reduced mammographic sensitivity, as dense tissue and tumors both appear radiopaque [234, 235]. Sensitivity drops from 85.7% in fatty breasts to 61% in extremely dense ones [236]. Supplemental imaging modalities, including DBT, ultrasound, and particularly MRI, improve detection rates [237]. However, dense breast alone is an imperfect predictor of benefit. Therefore, breast density should be incorporated with age and other risk factors into multivariable models to develop risk‐based screening strategies for high‐risk women [238].

Moderate risk factors also contribute meaningfully to overall risk. Racial and ethnic background plays a role: Ashkenazi Jewish women carry a higher prevalence of *BRCA* mutations, while breast cancer incidence is higher among Black women before the age of 45 years but higher in White women thereafter; Black women also have poorer survival outcomes [239, 240, 241]. Reproductive and hormonal factors such as early menarche, late menopause, nulliparity, oral contraceptive use, and postmenopausal hormone replacement therapy influence risk, though effects vary by formulation and individual susceptibility [242, 243, 244, 245]. Lifestyle‐related exposures, including elevated BMI, physical inactivity, alcohol consumption, and night‐shift work, further modify risk [246, 247, 248]. While moderate‐risk factors individually confer smaller effects than high‐risk factors, their cumulative interaction necessitates comprehensive risk assessment in clinical practice.

Research on traditional breast cancer risk factors has been progressively refined and expanded. Genetic studies have extended beyond *BRCA1/2* to include moderate‐ and low‐penetrance genes [249], with increasing emphasis on variant‐specific and locus‐specific functional effects rather than entire genes [250]. Hormonal influences have also been delineated with greater precision, particularly regarding hormone replacement therapy, where both dosage and duration critically shape risk [251]. Breast density, once assessed as a static measure, is now modeled through longitudinal trajectories to better reflect dynamic risk profiles [252]. Advances in imaging biomarkers further highlight this shift toward precision. Beyond density, quantitative radiologic features can provide additional risk‐relevant information. Parenchymal textural complexity has been demonstrated to improve prediction and model discrimination. Elevated background parenchymal enhancement on MRI corresponds to a 74% higher risk of breast cancer [253], while ultrasound‐based quantification of glandular tissue independently predicts risk and correlates with histological lobular involution [254]. Together, these findings expand both the breadth and depth of understanding, underscoring the value of moving from broad indicators toward more nuanced, functionally informed risk determinants.

### 4.2 Classical Risk Assessment Models

Several models have been developed to estimate breast cancer risk by integrating clinical, genetic, and lifestyle factors. The GAIL model, first reported in 1989 and later revised with Surveillance Epidemiology and End Results (SEER) and Breast cancer prevention trial (BCPT) data, remains widely used because it incorporates easily measured risk factors. However, it excludes genetic susceptibility such as *BRCA* mutations and is thus unsuitable for mutation carriers [213, 255, 256]. To address this, BRCAPRO and BOADICEA incorporated *BRCA* status, improving prediction for women with pathogenic variants [257, 258]. The IBIS (Tyrer‐Cuzick) model further advanced this approach by combining genetic and nongenetic factors, making it applicable to both carriers and noncarriers [259]. With breast density recognized as an independent risk factor, the breast cancer stem‐cell model integrated this feature demonstrated improved calibration across racial and ethnic groups. Independent validation using the ProF‐SC cohort found that BOADICEA and IBIS, which capture multigenerational family history, achieved superior predictive performance (area under the curve [AUC] 0.71 and 0.70). The findings also suggest that hybrid models incorporating polygenic risk alongside nonfamilial factors may further enhance risk stratification [260].

### 4.3 Exploration of New Risk Assessment Tools

PRS assesses an individual's susceptibility to certain diseases by describing the combined risk of many risk‐inducing single‐nucleotide polymorphisms (SNPs). It is an estimate of an individual's genetic susceptibility to developing complex diseases such as breast cancer. A current hot topic of exploration is the integration of PRS scores into existing models. For example, the IBIS model integrates PRS based on 18 SNPs associated with breast cancer and mammography density [261]. The BOADICEA model integrates a PRS based on 313 SNPs [262]. A study performed a comparative prospective validation of these two models and found that the extended BOADICEA model improved discrimination modestly in younger women (AUC 69.7 vs. 69.1%) and substantially in older women (AUC 64.6 vs. 56.8%), which is more accurate than the extended IBIS model in the European‐ancestry population [263]. A well‐documented concern is the limited applicability of current PRS models across populations of different genetic ancestries, largely due to the under‐representation of non‐European cohorts in breast cancer genome‐wide association studies [264, 265]. Population‐specific PRS developed in African American women have demonstrated markedly improved predictive performance compared with models derived from European datasets, underscoring the need for ancestry‐specific calibration [266]. Ongoing efforts aim to leverage multiethnic data to construct breast cancer risk prediction models with race‐specific adjustments to enhance their utility in non‐European populations; however, these approaches still require validation in prospective studies [267].

Deep learning‐ and AI‐based population screening models represent promising complementary strategies [268]. One advantage of AI models lies in their capacity to integrate diverse data types and automatically capture nonlinear relationships and latent interactions among variables, thereby enhancing discriminatory accuracy. For example, a deep learning model based on senescent nuclear morphology predicted future breast cancer risk from benign breast biopsy samples with greater accuracy than the Gail model [269]. Certain AI models also demonstrate independent predictive power. Mirai, an imaging‐based AI model, can predict 5‐year breast cancer risk from as little as a single screening mammogram, performing comparably even in the absence of additional clinical risk factors [268, 270]. In retrospective analyses, Mirai‐guided criteria for recommending supplemental MRI outperformed existing guidelines based on Tyrer‐Cuzick lifetime risk [268]. These findings highlight the ability of AI to extract biologically relevant information from raw data that conventional models cannot capture, offering the potential for widespread and equitable improvements in delivering breast cancer care.

## 5 Breast Cancer Screening

Breast cancer screening is undergoing a major transition from population‐based, uniform protocols toward more individualized, risk‐adapted strategies. This section reviews the full landscape of contemporary and emerging screening approaches and discuss the challenges of modern breast cancer screening, highlighting pathways toward more precise and equitable early detection.

### 5.1 Screening Methods for Breast Cancer

**FIGURE 3:**
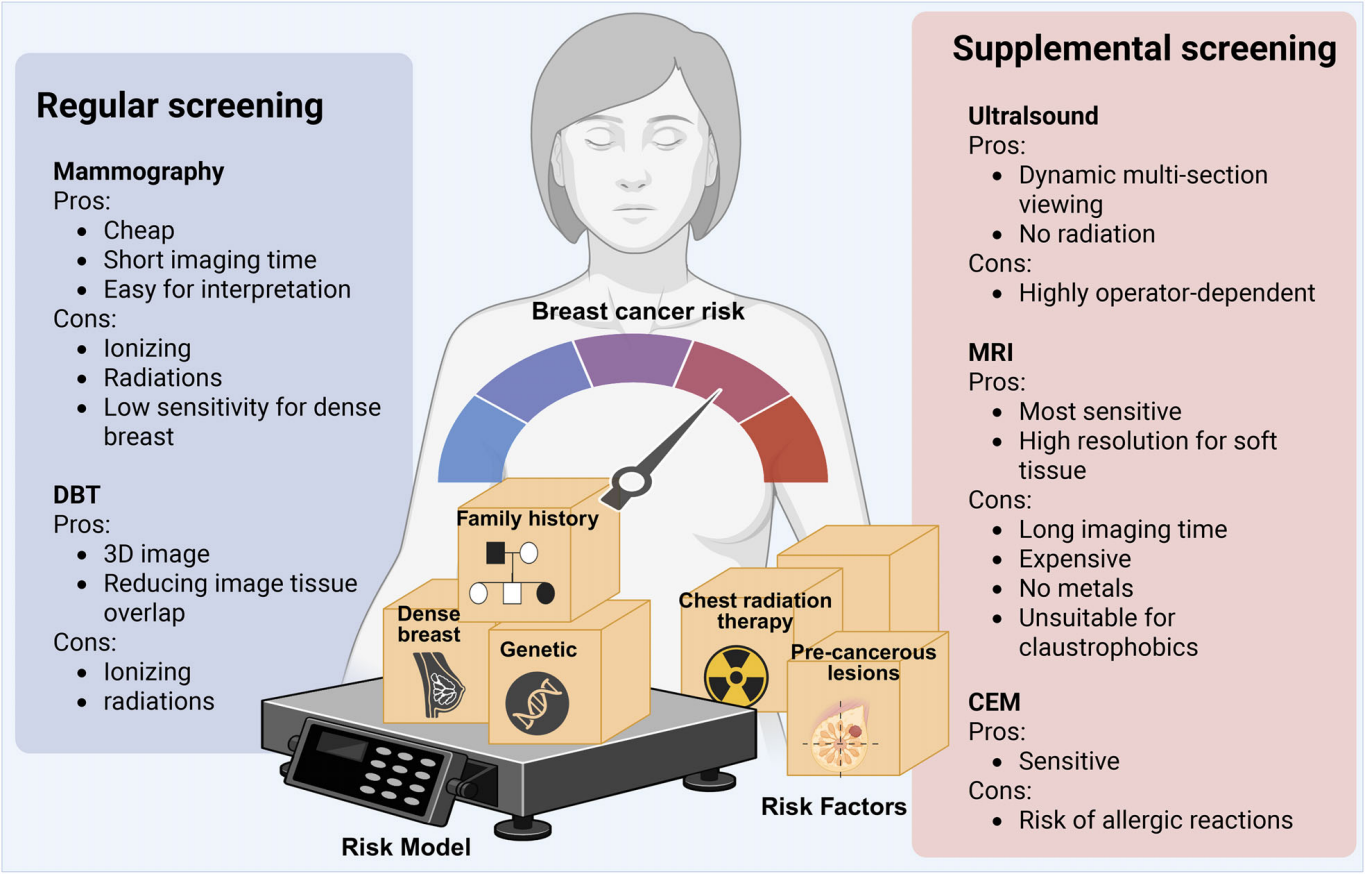
Illustration of risk‐based breast cancer screening. This paradigm outlines the stratification of individuals into regular or supplemental screening pathways based on the assessment of inherent and clinical risk factors.

The primary objective of breast cancer screening is to maximize early detection while minimizing the risks of false‐positive recalls, overdiagnosis, and unnecessary interventions. This section provides a detailed comparison of different imaging modalities, including mammography, DBT, ultrasound, MRI, and contrast‐enhanced mammography (CEM), with respect to diagnostic performance, clinical applicability, cost effectiveness, and potential harms. It further explores how these technologies are driving the transition of breast cancer screening from a “one‐size‐fits‐all” approach toward risk‐stratified, personalized strategies (Figure 3).

#### 5.1.1 Mammography: The Gold Standard With Limitations

Mammography has long been regarded as the gold standard in breast cancer screening. Large randomized controlled trials (RCTs) demonstrated that regular screening in women aged 50–70 years reduces breast cancer mortality by approximately 20% [271]. Its advantages include relatively low cost, broad accessibility, and well‐established standardized protocols.

Nevertheless, mammography has notable limitations, which have spurred the development of alternative modalities. In women with dense breasts, its sensitivity is reduced by 10–20%, resulting in a higher likelihood of missed cancers, particularly among younger women [272, 273]. The US FDA mandate to disclose breast density reflects growing recognition of these limitations and provides a rationale for supplemental screening [274]. High recall rates (6–27%) represent intrinsic harms that fuel debate over the balance of benefits and risks [275, 276, 277, 278]. The available data are not universally applicable. European countries generally report lower recall rates, ranging from 2 to 5% per screening round [279, 280, 281]. Differences in screening strategies, image‐reading approaches, and statistical methods may contribute to this variation. However, evidence suggests that these technical factors alone do not fully account for the observed disparities [282]. These regional differences underscore the need for context‐specific evidence when formulating international screening policies and harmonizing clinical guidelines. Estimates of breast cancer overdiagnosis associated with screening vary widely across studies, with reported rates ranging from approximately 1 to 50% [283, 284]. This variability reflects heterogeneity in estimation methodologies and population characteristics, emphasizing the need for caution when interpreting such data to avoid overestimating or misrepresenting the benefits of screening. However, regardless of the specific figures, the unnecessary treatments and psychological burden resulting from overdiagnosis remain a critical concern.

#### 5.1.2 DBT: A 3D Revolution

DBT addresses the tissue‐overlap limitations of conventional two‐dimensional mammography by reconstructing three‐dimensional images [26, 276]. Evidence shows clear incremental benefits in cancer detection and recall rates. In a large cohort analysis involving more than one million women, Conant et al. reported that DBT increased the detection rate from 4.5 to 5.3 cancers per 1000 women screened, while simultaneously reducing recalls [285]. This highlights that DBT's can provide dual advantage of improved sensitivity and reduced false positives.

Despite its superior diagnostic performance, DBT's role as a primary screening tool remains debatable. Some meta‐analyses suggest that DBT alone provides no additional benefit over standard mammography [286]. RCTs such as TOSYMA confirmed improved detection of invasive cancers with DBT, yet its long‐term clinical impact (e.g., on mortality reduction) and cost effectiveness remain to be established [287, 288]. Practical barriers also limit widespread adoption: DBT increases radiologists’ interpretation time and workload, and its equipment and training costs are significantly higher than those for conventional mammography, the challenges that are particularly evident in resource‐limited setting [289, 290].

#### 5.1.3 Ultrasound: A Complementary Tool for Dense Breasts

Ultrasound, free from ionizing radiation, is an appealing option for women with dense breasts and is particularly valuable in detecting small invasive cancers that mammography may miss [291, 292]. The J‐START trial demonstrated that combining ultrasound with mammography significantly improved sensitivity (91.1 vs. 77.0%), especially in women with dense breasts [293, 294]. However, this gain in sensitivity comes at the expense of lower specificity (87.7 vs. 91.4%), resulting in substantially higher false‐positive rates, unnecessary biopsies, patient anxiety, and increased healthcare costs [291, 295]. Moreover, ultrasound is highly operator dependent, leading to reduced reproducibility. Automated breast ultrasound partially addresses this limitation, but its broader adoption is constrained by cost [296].

#### 5.1.4 MRI: High Sensitivity for High‐Risk Women

MRI offers the highest sensitivity of all imaging modalities, particularly in women at elevated risk, such as *BRCA* mutation carriers. It detects cancers that mammography may miss [297]. However, its widespread use is hindered by high costs, lengthy examination times, and high false‐positive rates, making it unsuitable for population‐wide screening [26]. Abbreviated MRI was developed to overcome these barriers, substantially reducing scanning time while maintaining diagnostic performance comparable to full‐protocol MRI, thereby offering potential advantages in cost effectiveness and accessibility [298, 299, 300]·.

#### 5.1.5 CEM: A Hybrid Approach

CEM combines the accessibility of mammography with the contrast‐enhancement properties of MRI, offering a pragmatic middle ground. Studies suggest that CEM lowers false‐positive recalls and biopsy rates compared with standard MRI, with shorter acquisition and interpretation times [301]. This makes CEM a promising alternative for selected groups, such as high‐risk women with dense breasts. Nonetheless, its cancer detection rate and sensitivity remain lower than MRI [301], and further data are needed to clarify long‐term outcomes and evaluate risks associated with contrast agent exposure [302, 303].

#### 5.1.6 The Future of Noninvasive Testing

One of the most innovative developments in breast cancer screening is the analysis of volatile organic compounds (VOCs) in exhaled breath. VOCs, which are generated through cancer‐specific metabolic processes, can be detected using specialized analytical platforms, offering a fully noninvasive screening tool. Early clinical studies have reported sensitivity and specificity of 89.2% (222/249) and 87.7% (1355/1545), respectively, outperforming conventional mammography and ultrasound diagnostics [304]. Liquid biopsy approaches based on DNAme profiles from accessible mucosal samples are also emerging as promising tools. A recent large‐scale study systematically compared multiple noninvasive sample types and demonstrated that DNAme classifiers from cervical (AUC 0.75) and buccal (AUC 0.66) swabs markedly outperformed blood‐based assays (AUC 0.51) for breast cancer detection. Notably, DNAme alterations in these surrogate tissues closely mirrored those in breast tissue (AUC > 0.88), underscoring their potential application in multicancer early detection platforms that could be integrated into routine gynecological or dental practice [305, 306]. Despite these encouraging findings, such noninvasive technologies remain largely experimental, with limited application in clinical practice and a scarcity of robust validation data. Well‐designed, large‐scale prospective trials across diverse populations are essential to confirm their accuracy, reproducibility, and clinical utility before widespread implementation.

Breast cancer screening is thus evolving from reliance on a single “gold standard” toward more diversified and personalized approaches. Mammography remains the cornerstone but should be tailored according to age and breast density; for women with dense breasts, DBT or ultrasound may serve as valuable adjuncts; and for individuals at very high risk, such as carriers of pathogenic germline mutations, MRI remains the modality of choice. Emerging technologies such as MRI and CEM may provide new opportunities to balance diagnostic accuracy with cost effectiveness.

Future research should move from isolated comparisons of screening modalities to integrated, risk‐adapted models that account for family history, genetic susceptibility, and breast density. Such models, grounded in comprehensive evaluations of each modality's strengths and limitations, hold promise for maximizing the benefits of early detection while minimizing unnecessary harms and healthcare resource utilization, thereby advancing precision and efficiency in breast cancer screening.

### 5.2 Current Challenges in Breast Cancer Screening

#### 5.2.1 The Starting Age of Breast Cancer Screening

Most current guidelines recommend age‐based initiation of breast cancer screening for women at average risk, though the specific threshold varies widely (Table 3). A central controversy concerns whether mammography should begin at the age of 40 or 50 years [307, 308, 309, 310, 311, 312, 313].

**TABLE 3:** Summary of breast cancer screening recommendations in average‐risk women.

Organization	Published year	Country/region	References	Age range for screening	Screening methods for different age group	Screening intervals
AGO	2025	Germany	[341]	(1) Carriers of pathogenic/likely pathogenic variants (PVs) in breast‐cancer risk genes; (2) women without PVs but with ≥5% 10‐year risk by certified model; (3) breast‐cancer patients meeting GC‐HBOC germline testing criteria and diagnosed before the age of 46 years.	50–75: MAM	NA
EMSO (Pan‐Asian)	2024	China, Indonesia, India, Japan, Korea, Malaysia, Philippines, Singapore, Taiwan, and Thailand	[215, 216]	40–74	40–44: MAM	Regular may
					45–69: MAM	Biennial
					70–74: MAM	Regular may
CACA	2024	China	[214]	40–69	MAM ± US	Annual or biennial
				>70	MAM	Annual or biennial
USPSTF	2024	United States	[342]	40–74	MAM	Every other year
EMSO	2023	Europe	[215]	45–74	45–49: MAM	Regular may
					50–69: MAM	Biennial
					70–74: MAM	Regular may
ACR	2023	United States	[343]	≥40	40–44: MAM	Switch to a MAM every other year
					45–54: MAM	Annual
					≥55: MAM	Switch to MAM every year, or Annual
BH	2023	Bosnia and Herzegovina (BiH)	[218]	≥40	MAM	<55: annual; ≥55: biennial at least, or annually
CBR, SBM, and FEBRASGO	2023	Brazilian	[219]	≥40	MAM ± DBT	Annual
ECIB	2020	Europe	[344]	45–74	45–49: MAM	Either biennial or triennial mammography over annual screening
					60–69: MAM	Against annual mammography screening (strong); biennial mammography screening over triennial mammography screening (conditional)
					70–74: MAM	Against annual mammography screening (strong); triennial mammography screening over biennial mammography screening (conditional)
NCC	2020	China	[220]	≥45	MMA, US	Annual or biennial

The USPSTF 2023 revised its draft recommendations by lowering the starting age of breast cancer screening from 50 to 40 years. This decision was made by accumulated evidence showing meaningful benefits, although debate persists. Long‐term findings from the UK Age Trial, involving 160,000 women, showed that annual screening beginning at the age of 40 years reduced mortality by 25% compared with initiation at 50 years of age, with benefits persisting over two decades despite some attenuation [314]. Retrospective analyses from Canada further demonstrated higher 10‐year survival in women aged 40–49 years living in regions with organized screening compared with those without [3].

Younger women often present with aggressive, hormone‐driven tumors that progress more rapidly, making early detection particularly valuable [3, 315, 316]. Modeling studies also highlight disparities, showing that Black women experience higher mortality than White women under the same biennial protocols, and that expanding screening to ages 40–74 years yields a comparable benefit‐to‐harm ratio for Black women as starting at 50 years of age in White women [317]. Our group performed population‐based model analysis and similarly found that Black women reach risk thresholds earlier than White women, supporting race‐adapted screening initiation [318].

Nonetheless, earlier screening remains controversial. Women aged 40–49 years experience a high rate of false positive (estimated at 12%), leading to anxiety, biopsies, and overtreatment [319]. Dense breast tissue further reduces mammographic accuracy in this group. Although DBT increases detection of small lesions, it may also heighten overdiagnosis, especially in younger women [320]. Critics further note that observed survival gains may stem more from advances in systemic therapies than from screening itself, as European mortality declines were greatest in women under 50 regardless of screening policies [313, 321, 322].

Considering the resource burden of expanding eligibility, universal early screening risks diverting funds from interventions with clearer population‐level benefits [323]. Taken together, current evidence supports lowering initiation age, but the magnitude of benefit is modest and varies across subgroups. A risk‐adapted, rather than uniform, approach may better optimize outcomes.

#### 5.2.2 The Stopping Age of Breast Cancer Screening

Screening in older women presents the opposite dilemma. While the potential benefits of mammography accrue over years, harms such as false positives and overtreatment are immediate [324, 325]. Life expectancy and comorbidities are therefore critical in weighing net value [326]. Modeling studies suggest optimal stopping ages decline with increasing comorbidity, approximately 76, 74, 72, and 66 years for women with none, mild, moderate, and severe comorbidities, respectively, yielding benefit–harm balances similar to average‐risk women ceasing at 74 years of age. Yet guideline recommendations remain inconsistent: some specify 70 or 75 years of age, while others offer no explicit cutoff (Table 3) [327].

The evidence base is weak, as women >75 years are rarely included in randomized trials [327]. Observational and modeled studies report mixed findings that some suggest mortality reductions in women aged 70–74 years who continued screening, while benefits disappear beyond 75 years of age [325,^328^, ^329^, ^330^, ^331^, ^332^]. At the same time, harms escalate, with overdiagnosis estimated at 31% in women aged 70–74 years, 47% in those 75–84 years, and more than 50% in women over 85 years [325,^326^, ^330^, ^333^, ^334^]. False positives, unnecessary biopsies, and detection of indolent DCIS add further burden. New modalities such as DBT have not shown consistent benefit in elderly populations, and the exclusion of older women from clinical trials exacerbates uncertainty [335]. Despite this, many patients and clinicians remain supportive of continued screening, partly because harms are seen as less consequential than late‐stage breast cancer [324, 336].

In this context, personalized decision‐making is essential. Shared decision‐making models that integrate life expectancy, comorbidity, functional status, and patient preferences can guide individualized cessation. Risk‐adapted approaches that incorporate genetic susceptibility or prior cancer history may further refine decisions [337, 338]. Such patient‐centered frameworks are more appropriate than rigid chronological thresholds.

#### 5.2.3 Screening Frequency

The frequency of breast cancer screening remains unsettled. Most guidelines recommend annual or biennial intervals for women aged ≥40 years, with some stratification by age (Table 3).

Observational data from more than 8000 patients demonstrated that biennial or irregular screening was associated with later‐stage presentation and 42% higher mortality compared with annual screening, with consistent benefits across age, race, and menopausal status [339]. A systematic review of randomized, observational, and modeling studies concluded that biennial screening achieves a favorable benefit–harm balance for women aged 50–69 years, whereas women aged 45–49 years may benefit more from annual intervals. For women aged ≥70 years, findings are inconsistent, with some suggesting reduced harms with longer intervals but limited survival impact [340]. However, the certainty of evidence is low due to a paucity of randomized data directly comparing frequencies. Cost‐effectiveness analyses in different healthcare settings are needed to clarify optimal strategies.

#### 5.2.4 Global Inequities in Breast Cancer Screening

**FIGURE 4:**
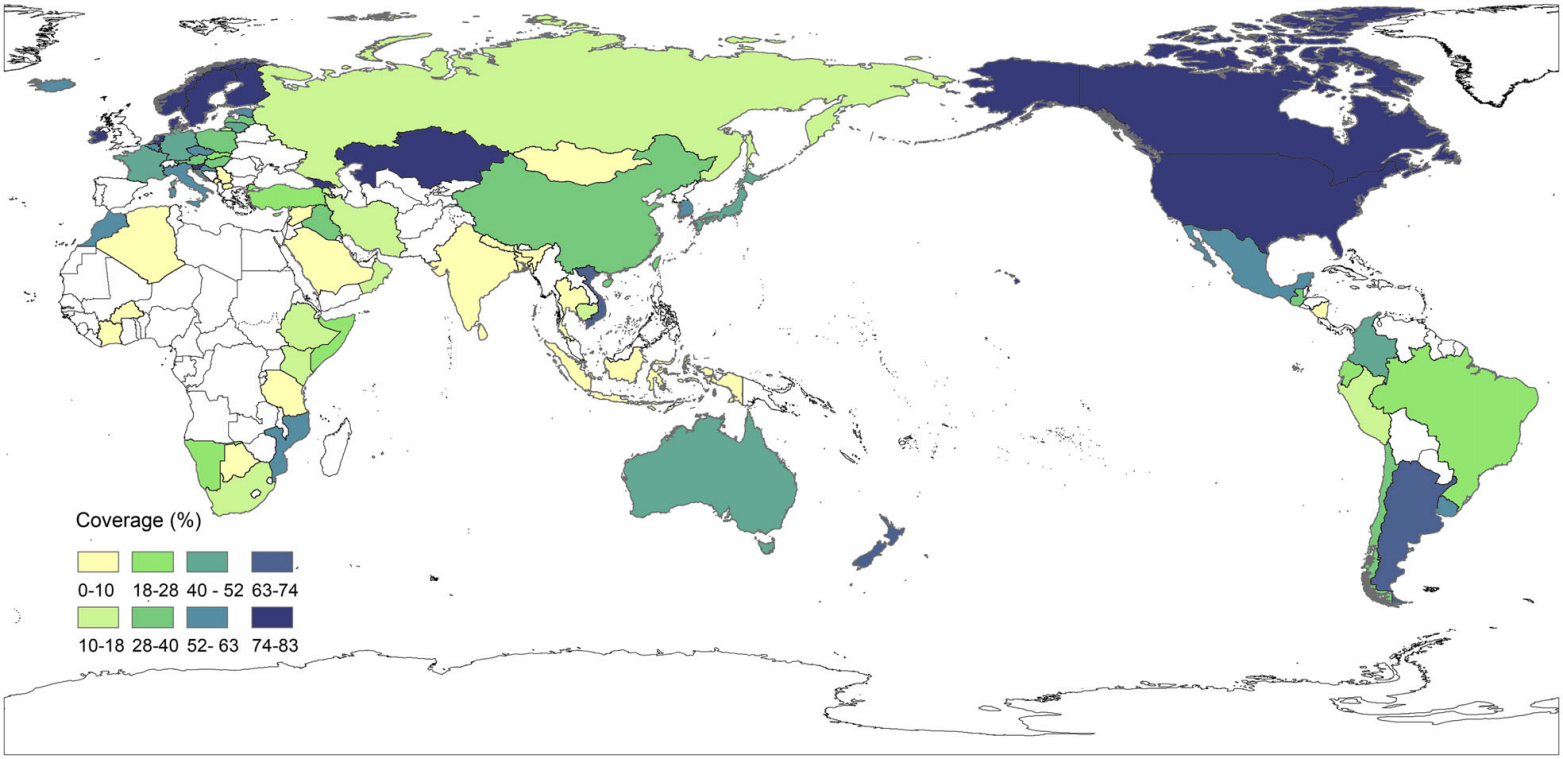
Global breast cancer screening program coverage. The world map depicts disparities in screening program coverage across different countries. [Map approval number GS (2016) 1665.]

Screening coverage is highly uneven worldwide. By 2021, 63% of WHO member states/regions reported national screening strategies [data from the Cancer Screening in Five Continents (CanScreen5) database, accessed on July 24, 2025]. The coverage ranged from 83% in Denmark to just 0.25% in North Macedonia (Figure 4 and Table S1). These disparities reflect differences in infrastructure, resource allocation, socioeconomic development, awareness, and cultural attitudes.

High‐income countries such as the United States, the United Kingdom, and much of Northern Europe countries operate mature, organized programs with regular mammography for women aged 40–75 years, contributing to early detection and mortality reductions. By contrast, many low‐ and middle‐income countries face major barriers. For example, Singapore offers biennial mammography for women aged 50–69 years, while Indonesia, Thailand, and Vietnam lack organized programs; in Thailand, clinical breast examination often substitutes for mammography, reflecting resource limitations [345].

Within‐country inequities also persist. In China, screening participation rose from 20% in 2014 to over 50% in 2022, yet regional variation remained stark: Beijing reported coverage above 80%, whereas central and western provinces lagged under 40%. Rural participation has consistently trailed urban uptake. Such disparities translate into later‐stage diagnoses and poorer outcomes for disadvantaged populations [346].

To address these gaps, the WHO's Global Breast Cancer Initiative aims to reduce mortality by 2.5% annually, emphasizing early detection and standardized treatment, particularly in low‐ and middle‐income countries. The initiative promotes context‐appropriate strategies such as ultrasound or clinical breast examination, alongside investment in primary healthcare capacity and public health education. These efforts, if implemented equitably, could narrow disparities and improve global outcomes.

### 5.3 Promising Strategies for the Future

Risk‐adapted screening offers a potential pathway to reconcile conflicting evidence on initiation, cessation, and frequency. A multicenter Chinese cohort study demonstrated that women screening at 43, 48, and 55 years of age (high, intermediate, and low risk, respectively) reached mortality risks equivalent to average‐risk women starting at the age of 50 years. Such approaches optimize allocation of limited resources by targeting those most likely to benefit [347]. Interval stratification may further refine the efficiency. A predictive model developed by Kerlikowske and colleagues identified 12% of women who derived clear benefit from annual versus biennial screening, while most women did not. For average‐ and high‐risk women whose outcomes were not influenced by frequency alone, alternative modalities may be necessary to improve efficacy [348].

Collectively, these findings underscore the limitations of one‐size‐fits‐all policies. Moving toward individualized screening strategies—integrating genetic predisposition, race and ethnicity, health status, and comorbidities—offers the most promising route to maximizing benefits, minimizing harms, and addressing inequities in global breast cancer control.

## 6 Prevention

Breast cancer is associated with both modifiable and nonmodifiable risk factors. Modifiable risks factors, such as BMI, alcohol consumption, and physical activity, can be mitigated through lifestyle changes, whereas nonmodifiable risk factors of genetic susceptibility, breast density, and prior radiation exposure remain largely irreversible. Preventive strategies have been developed to lower cancer risk in high‐risk populations. Table 4 summarizes current guideline recommendations for preventive interventions.

**TABLE 4:** Summary of preventive inventions for high‐risk women.

Organization	Reference	Published year	Country/region	Aimed population	Interventions
NCCN	[213]	2025	US	Individuals with BRCA1/2 pathogenic/likely pathogenic germline variants (and other hereditary high‐risk gene carriers)	Risk‐reducing mastectomy and enhanced breast MRI surveillance
ESMO	[349]	2025	Europe	Unaffected carriers of germline pathogenic variants in high‐penetrance BC genes	Risk‐reducing bilateral mastectomy (RRBM) and enhanced breast MRI surveillance
AIOM	[350]	2025	Italian	BRCA1/2 germline pathogenic‐variant carriers, especially women already affected by breast cancer and at high contralateral/ovarian cancer risk	Bilateral risk‐reducing mastectomy and/or salpingo‐oophorectomy
EMSO (Pan‐Asian)	[215, 216]	2024	China, Indonesia, India, Japan, Korea, Malaysia, Philippines, Singapore, Taiwan and Thailand	High‐risk women with DCIS history	Tamoxifen or AIs
BH	[218]	2023	Bosnia and Herzegovina (BiH)	BRCA1, BRCA2, PALB2, PTEN, and TP53 mutation carriers	Consider bilateral prophylactic mastectomy

### 6.1 Risk‐Reducing Medications

Randomized trials have demonstrated that some medications, including SERMs, such as tamoxifen and raloxifene, and AIs, such as anastrozole and exemestane, can reduce the risk of breast cancer [351, 352, 353, 354, 355]. A meta‐analysis reported risk ratios (RR) of 0.69 [95% CI 0.59–0.84] for tamoxifen, 0.44 [95% CI 0.24–0.80] for raloxifene, and 0.45 [95% CI 0.26–0.70] for AIs (anastrozole and exemestane) compared with placebo [356]. The STAR trial demonstrated that tamoxifen was more effective than raloxifene in reducing long‐term breast cancer risk, but raloxifene was associated with fewer adverse events (RR = 0.55, 95% CI 0.36–0.83 for endometrial cancer, RR = 0.19, 95% CI 0.12–0.29 for uterine hyperplasia, and RR = 0.75, 95% CI 0.60–0.93 for thromboembolic events) [357]. To date, no prospective head‐to‐head trials directly comparing SERMs and AIs have been conducted, leaving uncertainty regarding relative efficacy and safety. Of note, tamoxifen is currently recommended only for premenopausal women, reflecting the lack of robust data in this population [358].

Despite their proven clinical benefits, risk‐reducing medications remain underutilized [359]. A major barrier is women's concern about side effects, including thromboembolic events, endometrial cancer, cataracts, vasomotor symptoms, and musculoskeletal complaints [356, 360, 361]. Prior to the use of risk‐reducing medications, it is imperative that the risk of disease in women be evaluated in order to ascertain which women are at elevated risk. However, it is challenging for primary care providers to identify women who are eligible for treatment due to a lack of familiarity with risk prediction models, limited time, insufficient information about risk reduction programs, inadequate counselling and training, and insufficient reimbursement [362]. The accuracy of existing risk models is also suboptimal. A meta‐analysis of 25 studies involving over 5 million women found that most methods achieved AUCs of only 0.55–0.65, limiting clinical utility [356]. Emerging evidence suggests that biomarkers, such as estradiol‐to‐sex hormone‐binding globulin ratios, may help refine the prediction of chemo‐preventive efficacy, but such approaches require validation [363]. Nevertheless, the gap between clinical evidence and real‐world uptake highlights the need for developing more precise risk assessment tools and strategies to overcome the barriers at both patient and provider levels.

### 6.2 Risk‐Reducing Mastectomy

Risk‐reducing mastectomy (RRM), also known as prophylactic mastectomy, has three commonly used procedures, including simple (total) mastectomy, skin‐sparing mastectomy, and nipple‐sparing mastectomy, which is usually followed by breast reconstruction [364]. A meta‐analysis showed a 90% reduction in breast cancer risk in women after RRM [365]. However, residual risk remains, and surgical morbidity, such as wound complications and infections, must be carefully weighed. Women with high‐risk germline mutations or prior chest irradiation before the age of 30 years are most likely to benefit from RRM [364].

For *BRCA1/2* carriers, risk‐reducing salpingo‐oophorectomy (RRSO) is additionally recommended after childbearing to reduce ovarian and breast cancer risk. Prospective data demonstrate that RRSO substantially lowers the incidence of *BRCA*‐related cancers (HR = 0.25, 95% CI 0.08–0.74) [366]. Menopause symptoms, such as osteoporosis, cardiovascular disease, changes to vasomotor symptoms, sleep disturbances, and sexual discomfort, are major side effects of RRSO due to the reduction of estrogen and progesterone production [367]. Hormone replacement therapy alleviates these symptoms and does not appear to diminish the protective effect of RRSO [368]. Estrogen‐only regimens may be associated with the lowest breast cancer risk [369].

Most studies indicate that RRM and RRSO reduce cancer‐related anxiety without significantly impairing health‐related quality of life [367]. Nevertheless, the invasive nature of these procedures necessitates a nuanced discussion of benefits, risks, reconstructive options, and menopausal management, ensuring that patients’ informed choices are grounded in both oncologic outcomes and long‐term quality‐of‐life considerations.

## 7 Perspectives

Building on these advances, it is equally important to consider how emerging discoveries may shape the future trajectory of breast cancer research and care. The following sections explore several key opportunities and challenges that are likely to shape ongoing efforts in the field.

### 7.1 Multiomic Integration for Precision Oncology

We are transitioning to an era of multidimensional multiomic integration, where emerging technologies such as spatial transcriptomics are unveiling tumor biology at unprecedented depth. Traditional breast cancer research has largely relied on histopathology and bulk sequencing, which provide only averaged molecular information at the tissue level and fail to capture the cellular heterogeneity within tumors. This heterogeneity is a key driver of therapeutic failure and resistance. The advent of scRNA‐seq has provided a powerful tool to address this limitation [370]. By enabling transcriptomic profiling at the single‐cell level, scRNA‐seq has illuminated mechanisms of therapeutic resistance and clonal evolution in breast cancer [371, 372]. Spatial transcriptomics, in turn, represents a transformative advance. It complements scRNA‐seq by preserving the spatial context of cells within native tissue and thus overcoming the loss of information about cellular proximity and interactions [373, 374]. This capability has revealed dynamic interactions and spatial patterns among tumor, immune, and stromal cells within the TME at an unprecedented resolution [375, 376], offering critical insights into why some patients respond to immunotherapy while others do not.

Multiomic analyses allow systematic interrogation across biological layers, but they also pose the formidable challenge of extracting clinically meaningful insights from exponentially expanding datasets. AI has demonstrated considerable potential in integrating multimodal oncologic data streams [377]. AI plays a pivotal role in enabling precision medicine by predicting patient‐specific therapeutic responses based on genomic, microenvironmental, or imaging features, thereby facilitating optimal treatment selection before therapy initiation. Generative AI is increasingly applied across the drug development pipeline, accelerating the design and screening of novel targeted agents and antibody therapeutics [378]. Deep learning further enables automated extraction of radiomic features from digital pathology and imaging data, shifting from purely morphological assessments toward functional and prognostic predictions [270]. Nevertheless, improving model interpretability and bridging the gap from algorithmic development to clinical application remain urgent priorities.

### 7.2 Combining Data‐Driven and Hypothesis‐Driven Research

Unlike the traditional hypothesis‐driven paradigm, which proceeds “from question to data,” data‐driven research leverages large‐scale datasets to identify patterns and generate novel hypotheses that can subsequently be validated through rigorous experimentation. This new paradigm has been powered by the accumulation of massive datasets deposited in large public repositories. A notable example is the analysis of breast cancer data from The Cancer Genome Atlas, which identified key prognostic factors and provided new directions for mechanistic studies and therapeutic target discovery [379, 380]. Future research will increasingly require the integration of diverse data types that extend beyond conventional clinical phenotypes, encompassing molecular profiles, digital pathology images, socioeconomic variables, and lifestyle factors [381, 382]. Achieving this vision necessitates multidisciplinary collaboration among clinicians, experimental scientists, biostatisticians, and machine learning experts, with data sharing and standardization as central challenges. Major obstacles include missing data, inconsistent formats, privacy concerns, and institutional barriers [383]. In addition, global data equity remains a pressing issue. Large‐scale databases are currently dominated by populations of European ancestry, with limited representation of non‐European populations [384]. Establishing ethnically and racially diverse databases and accounting for population diversity and data bias from the outset are essential to ensure that future research advances can benefit all patients equitably.

## 8 Summary

Breast cancer is the most common cancer and the leading cause of cancer‐related death among women worldwide. Early detection and the implementation of precision treatment strategies are essential for improving clinical outcomes. This review highlights recent progress in molecular characterization, targeted therapy development, surveillance approaches, and precision prevention. A deeper understanding of the pathogenic mechanisms underlying breast cancer is critical for informing novel therapeutic strategies and refining monitoring and prevention efforts. Targeted treatments tailored to specific molecular features now play a significant role in precision oncology. The integration of endocrine therapy, HER2‐directed agents, CDK4/6i, PI3K/AKT/mTOR pathway inhibitors, ADCs, and immunotherapies has profoundly expanded treatment options, improving outcomes across distinct molecular subtypes. Equally important, advances in screening and surveillance have strengthened the capacity for early detection, translating into better prognosis and reduced mortality. Improvements in diagnostic technologies, particularly imaging, have further enhanced accuracy and decreased both misdiagnosis and underdetection. Risk‐reducing interventions, including preventive surgery and medications, exhibit significant protective benefit for high‐risk carriers in clinical practice, albeit with nuanced considerations of surgical morbidity and long‐term quality of life. These developments collectively underscore the shift from one‐size‐fits‐all approaches toward highly individualized management. Yet, despite remarkable progress, challenges such as therapeutic resistance, toxicity, cost effectiveness, and global inequities in access persist, highlighting the needs of developing a balanced appraisal covering innovation and real‐world applicability.

Looking ahead, the convergence of multiomic technologies, spatial biology, and AI is expected to broaden the possibilities for breast cancer research and clinical care. These emerging approaches enable detailed characterization of tumor heterogeneity at single‐cell and spatial resolution. Coupled with genomic, transcriptomic, proteomic, radiomic, pathomic, and clinical datasets, they may open new avenues for understanding therapeutic resistance, identifying potential targets, and advancing predictive biomarker development. Importantly, the full realization of these advances will depend on robust data‐sharing frameworks, increased representation of diverse populations, and effective translation from algorithmic models to bedside decision‐making. As breast cancer research advances toward a more integrated, data‐driven paradigm, it will be important to translate scientific insights into practical applications while maintaining an appropriate balance between technological innovation and patient‐centered care. Through such efforts, precision medicine may continue to evolve in ways that benefit individuals at risk for or living with breast cancer, helping ensure that scientific progress ultimately supports improved care for all.

## Author Contributions

Conceptualization, data curation, figures drawing, writing – original draft: Huijun Lei, Jinzhen Fu, and Wei Gu. Figures drawing, writing – original draft: Hongjin Qiao. Conceptualization, figures drawing, writing – review and editing: Huixue Guo, Zijian Chen. Writing – review and editing: San Ming Wang. Project administration, supervision, conceptualization, writing – review and editing: Tianhui Chen. All authors have read and approved the final manuscript.

## Funding

This work was supported by grants from National Key Research‐Development Program of China (2019YFE0198800), Key Research‐Development Program of Zhejiang Province (2017C03013), Ten‐Thousand Talents Plan of Zhejiang Province (2021R52020). The funding agencies had no role in the design and conduct of the study; collection, management, analysis, and interpretation of the data, preparation, review, or approval of the manuscript; and decision to submit the manuscript for publication.

## Ethics Statement

The authors have nothing to report.

## Conflicts of Interest

The authors declare no conflicts of interest.

## Supporting information




**Supporting Table 1**: Global breast cancer screening program coverage.

## Data Availability

The authors have nothing to report.
