# Supplementary material for: Breast Cancer: Molecular Pathogenesis, Targeted Therapy, Screening, and Prevention
Source: MedComm (2020). 2026 Jan 7;7(1):e70560. doi: 10.1002/mco2.70560 (PMC12778424; doi:10.1002/mco2.70560)
Supplement: Supplementary file 1 — Supporting Table 1: Global breast cancer screening program coverage. [file MCO2-7-e70560-s001.docx]

**Title: Breast Cancer: Molecular Pathogenesis, Targeted Therapy, Screening, and Prevention**

**Running title: Breast Cancer Treatment and Prevention**

Huijun Lei^1,2#^| Jinzhen Fu^1,3#^|Wei Gu^1,3#^|Hongjin Qiao^1,3^|Huixue Guo^1,4^|Zijian Chen^1,3^|San Ming Wang^2^|Tianhui Chen^1*^

^1^Department of Cancer Prevention, Zhejiang Cancer Hospital, Hangzhou Institute of Medicine (HIM), Chinese Academy of Sciences, Hangzhou, Zhejiang, China

^2^Faculty of Health Sciences, University of Macau, Taipa, Macau, China

^3^Postgraduate training base Alliance of Wenzhou Medical University (Zhejiang Cancer Hospital), Wenzhou, Zhejiang, China

^4^Hangzhou Normal University, Hangzhou, Zhejiang, China

***Correspondence:** Tianhui Chen, Department of Cancer Prevention, Zhejiang Cancer Hospital, Hangzhou Institute of Medicine (HIM), Chinese Academy of Sciences, Hangzhou, Zhejiang, China

E-mail: [chenth@zjcc.org.cn](mailto:chenth@zjcc.org.cn)

^#^These authors have contributed equally to this work as co-first authors.

Supplementary Table 1. Global breast cancer screening program coverage.

| **Country** | **Year** | **Coverage** | **Age** | **Coverage2** | **Age2** | **Reference** |
| --- | --- | --- | --- | --- | --- | --- |
| Belgium | 2021 | 55.80 | 50-69 |  |  | https://ec.europa.eu/eurostat/databrowser/view/HLTH_PS_PREV__custom_7978672/bookmark/ |
| Bulgaria | 2021 |  | 50-69 |  |  | https://ec.europa.eu/eurostat/databrowser/view/HLTH_PS_PREV__custom_7978672/bookmark/ |
| Czechia | 2021 | 59.00 | 50-69 |  |  | https://ec.europa.eu/eurostat/databrowser/view/HLTH_PS_PREV__custom_7978672/bookmark/ |
| Denmark | 2021 | 83.00 | 50-69 |  |  | https://ec.europa.eu/eurostat/databrowser/view/HLTH_PS_PREV__custom_7978672/bookmark/ |
| Germany | 2021 | 51.50 | 50-69 |  |  | https://ec.europa.eu/eurostat/databrowser/view/HLTH_PS_PREV__custom_7978672/bookmark/ |
| Estonia | 2021 | 58.70 | 50-69 |  |  | https://ec.europa.eu/eurostat/databrowser/view/HLTH_PS_PREV__custom_7978672/bookmark/ |
| Ireland | 2021 | 74.80 | 50-69 |  |  | https://ec.europa.eu/eurostat/databrowser/view/HLTH_PS_PREV__custom_7978672/bookmark/ |
| Greece | 2021 |  | 50-69 |  |  | https://ec.europa.eu/eurostat/databrowser/view/HLTH_PS_PREV__custom_7978672/bookmark/ |
| Spain | 2021 |  | 50-69 |  |  | https://ec.europa.eu/eurostat/databrowser/view/HLTH_PS_PREV__custom_7978672/bookmark/ |
| France | 2021 | 46.90 | 50-69 |  |  | https://ec.europa.eu/eurostat/databrowser/view/HLTH_PS_PREV__custom_7978672/bookmark/ |
| Croatia | 2021 | 56.00 | 50-69 |  |  | https://ec.europa.eu/eurostat/databrowser/view/HLTH_PS_PREV__custom_7978672/bookmark/ |
| Italy | 2021 | 55.90 | 50-69 |  |  | https://ec.europa.eu/eurostat/databrowser/view/HLTH_PS_PREV__custom_7978672/bookmark/ |
| Cyprus | 2021 | 22.69 | 50-69 |  |  | https://ec.europa.eu/eurostat/databrowser/view/HLTH_PS_PREV__custom_7978672/bookmark/ |
| Latvia | 2021 | 30.80 | 50-69 |  |  | https://ec.europa.eu/eurostat/databrowser/view/HLTH_PS_PREV__custom_7978672/bookmark/ |
| Lithuania | 2021 | 46.50 | 50-69 |  |  | https://ec.europa.eu/eurostat/databrowser/view/HLTH_PS_PREV__custom_7978672/bookmark/ |
| Luxembourg | 2021 | 53.90 | 50-69 |  |  | https://ec.europa.eu/eurostat/databrowser/view/HLTH_PS_PREV__custom_7978672/bookmark/ |
| Hungary | 2021 | 29.79 | 50-69 |  |  | https://ec.europa.eu/eurostat/databrowser/view/HLTH_PS_PREV__custom_7978672/bookmark/ |
| Malta | 2021 | 77.80 | 50-69 |  |  | https://ec.europa.eu/eurostat/databrowser/view/HLTH_PS_PREV__custom_7978672/bookmark/ |
| Netherlands | 2021 | 72.90 | 50-69 |  |  | https://ec.europa.eu/eurostat/databrowser/view/HLTH_PS_PREV__custom_7978672/bookmark/ |
| Austria | 2021 | 40.10 | 50-69 |  |  | https://ec.europa.eu/eurostat/databrowser/view/HLTH_PS_PREV__custom_7978672/bookmark/ |
| Poland | 2021 | 33.21 | 50-69 |  |  | https://ec.europa.eu/eurostat/databrowser/view/HLTH_PS_PREV__custom_7978672/bookmark/ |
| Romania | 2021 |  | 50-69 |  |  | https://ec.europa.eu/eurostat/databrowser/view/HLTH_PS_PREV__custom_7978672/bookmark/ |
| Slovenia | 2021 | 77.80 | 50-69 |  |  | https://ec.europa.eu/eurostat/databrowser/view/HLTH_PS_PREV__custom_7978672/bookmark/ |
| Slovakia | 2021 | 25.46 | 50-69 |  |  | https://ec.europa.eu/eurostat/databrowser/view/HLTH_PS_PREV__custom_7978672/bookmark/ |
| Finland | 2021 | 82.20 | 50-69 |  |  | https://ec.europa.eu/eurostat/databrowser/view/HLTH_PS_PREV__custom_7978672/bookmark/ |
| Sweden | 2021 | 80.00 | 50-69 |  |  | https://ec.europa.eu/eurostat/databrowser/view/HLTH_PS_PREV__custom_7978672/bookmark/ |
| Iceland | 2021 | 54.00 | 50-69 |  |  | https://ec.europa.eu/eurostat/databrowser/view/HLTH_PS_PREV__custom_7978672/bookmark/ |
| Liechtenstein | 2021 | 10.00 | 50-69 |  |  | https://ec.europa.eu/eurostat/databrowser/view/HLTH_PS_PREV__custom_7978672/bookmark/ |
| Norway | 2021 | 74.60 | 50-69 |  |  | https://ec.europa.eu/eurostat/databrowser/view/HLTH_PS_PREV__custom_7978672/bookmark/ |
| Switzerland | 2021 |  | 50-69 |  |  | https://ec.europa.eu/eurostat/databrowser/view/HLTH_PS_PREV__custom_7978672/bookmark/ |
| Montenegro | 2021 | 6.49 | 50-69 |  |  | https://ec.europa.eu/eurostat/databrowser/view/HLTH_PS_PREV__custom_7978672/bookmark/ |
| North Macedonia | 2021 | 0.25 | 50-69 |  |  | https://ec.europa.eu/eurostat/databrowser/view/HLTH_PS_PREV__custom_7978672/bookmark/ |
| Serbia | 2021 | 5.20 | 50-69 |  |  | https://ec.europa.eu/eurostat/databrowser/view/HLTH_PS_PREV__custom_7978672/bookmark/ |
| Türkiye | 2021 | 20.54 | 50-69 |  |  | https://ec.europa.eu/eurostat/databrowser/view/HLTH_PS_PREV__custom_7978672/bookmark/ |
| America | 2022 | 76.5 | 50-74 | 59.1 | 40-49 | https://www.cdc.gov/mmwr/volumes/73/wr/mm7315e1.htm#:~:text=In%202022%2C%20more%20than%20three,by%20state%20and%20sociodemographic%20characteristics. |
| Canada | 2024 | 79 | 50-74 |  |  | https://www150.statcan.gc.ca/n1/pub/82-625-x/2025001/article/00002-eng.htm#a7 |
| The United Mexican States | 2021 | 59.8 | 40-49 | 65.8 | 60-65 | https://www.ajol.info/index.php/gab/article/view/203341 |
| Nicaragua | 2019 | 3.5 | 40+ |  |  | https://canscreen5.iarc.fr/?page=countryfactsheet&q=GHA |
| República de Colombia | 2015 | 48.1 | 40-69 |  |  | PMID: 33611666 |
| República del Ecuador | 2018 | 23.8 | 50-69 |  |  | https://journals.co.za/doi/abs/10.10520/ejc-genbeh_v19_n3_a21 |
| Republic of Peru | 2018 | 16.9 | 40-59 |  |  | PMID: 31665129 |
| Republic of Chile | 2019 | 38.7 | 50-69 |  |  | https://canscreen5.iarc.fr/?page=countryfactsheet&q=GHA |
| Republic of Argentina | 2013 | 65.6 | 40-70 |  |  | https://sar.org.ar/wp-content/uploads/2023/09/Breast-Imaging-in-Argentina.pdf |
| The Federative Republic of Brazil | 2019 | 28.1 | 50-69 |  |  | PMID: 36595736 |
| Uruguay | 2019 | 52.9 | 50-69 |  |  | https://canscreen5.iarc.fr/?page=countryfactsheet&q=GHA |
| Morocco | 2019 | 56.2 | 40-69 |  |  | https://canscreen5.iarc.fr/?page=countryfactsheet&q=GHA |
| Mozambique | 2020 | 54.9 | 25-45 |  |  | https://canscreen5.iarc.fr/?page=countryfactsheet&q=GHA |
| The Burkina Faso | 2010 | 7.5 | 15-49 |  |  | PMID: 33046473 |
| The Republic of Côte d'Ivoire | 2011-2012 | 5.2 | 15-50 |  |  | PMID: 33046473 |
| The Republic of Kenya | 2014 | 13.6 | 15-51 |  |  | PMID: 33046473 |
| The Republic of Namibia | 2013 | 23.1 | 15-52 |  |  | PMID: 33046473 |
| The Islamic Republic of Iran | 2016 | 16.7 | 20-65 |  |  | PMID: 32460471 |
| Republic of Armenia | 2020 | 18.4 | 35-65 |  |  | PMID: 37145984 |
| Georgia | 2020 | 79 | 50-75 |  |  | https://www.cancerregistryeducation.org/Files/Org/f3f3d382a7a242549a9999654105a63b/site/Collaboration-2020_NPCR_GEORGIA_SUCCESS_STORY.pdf |
| The Republic of Kazakhstan | 2021 | 79.4 | 40-70 |  |  | DOI: 10.1016/j.annonc.2022.03.170 |
| India | 2020 | 5 | 40+ |  |  | PMID: 40108533 |
| The Socialist Republic of Viet Nam | 2019 | 63 | 30-74 |  |  | PMID: 35622840 |
| Bangladesh | 2019 | 1.7 | 30+ |  |  | https://canscreen5.iarc.fr/?page=countryfactsheet&q=GHA |
| Japan | 2017 | 47.4 | 40-69 |  |  | https://www.mhlw.go.jp/toukei/saikin/hw/k-tyosa/k-tyosa22/dl/04.pdf |
| Republic of Korea | 2016 | 56.7 | 40-79 |  |  | https://canscreen5.iarc.fr/?page=countryfactsheet&q=GHA |
| Nepal | 2022 | 6.5 | 30-49 |  |  | PMID: 38466682 |
| China | 2019 | 30.9 | 35-64 | 22.3 | 20+ | PMID: 37193086 |
| Singapore | 2016 | 38.6 | 50-69 |  |  | https://data.gov.sg/collections/520/view |
| The Kingdom of Thailand | 2010 | 4.5 | 45-59 |  |  | PMID: 28282430 |
| Republic of Indonesia | 2016 | 5.2 |  |  |  | https://www.marubeni.com/en/news/2023/info/00030.html |
| Australia | 2023 | 52 | 50-74 |  |  | https://www.aihw.gov.au/reports/cancer-screening/breastscreen-australia-monitoring-report-2024/summary |
| New Zealand | 2024 | 70 | 50-69 |  |  | PMID: 40718040 |
| Russia | 2009 | 18 | 40-60 |  |  | PMID: 22276049 |
| Tanzania | 2022 | 5 | 15-49 |  |  | PMID: 39485789 |
| Somalia | 2018 | 25 |  |  |  | https://www.mprnews.org/story/2018/12/26/program-seeks-to-persuade-somali-women-to-get-cancer-screenings |
| Botswana | 2016 | 6 | 15-64 |  |  | PMID: 34347841 |
| Kingdom of Saudi Arabia | 2015 | 8 | 35-60 |  |  | PMID: 35414623 |
| The Republic of Guatemala | 2020 | 36 | 40+ |  |  | PMID: 34196900 |
| South Africa | 2012 | 13.4 | 30-70 |  |  | PMID: 29936716 |
| The Sultanate of Oman | 2016 | 18 | 50-69 |  |  | https://digitalcommons.unmc.edu/cgi/viewcontent.cgi?article=1013&context=coph_slce |
| The Democratic Socialist Republic of Sri Lanka | 2012 | 3.6 | 35+ |  |  | PMID: 22799304 |
| Cambodia | 2022 | 10.6 | 15-49 |  |  | PMID: 39883702 |
| Mongolia | 2013 | 1.7 |  |  |  | PMID: 28176935 |
| Ethiopia | 2020 | 13.6 | 20-70 |  |  | PMID: 33447077 |
| The Republic of Iraq | 2022 | 35.1 |  |  |  | PMID: 36248755 |
| Algeria | 2016 | 7.6 | 18+ |  |  | PMID: 33333411 |
| The Syrian Arab Republic | 2019 | 5 | 20-89 |  |  | PMID: 38097985 |
